# Demographic, epidemiological, and health transitions: are they relevant to population health patterns in Africa?

**DOI:** 10.3402/gha.v7.22443

**Published:** 2014-05-15

**Authors:** Barthélémy Kuate Defo

**Affiliations:** Public Health Research Institute and Department of Demography, University of Montreal, Montreal, Quebec, Canada

**Keywords:** systematic review, demographic transition, epidemiological transition, health transition, population health, epidemiology of population change, global health, Africa, sub-Saharan Africa, cross-national comparison, sex differentials

## Abstract

**Background:**

Studies of trends in population changes and epidemiological profiles in the developing world have overwhelmingly relied upon the concepts of demographic, epidemiological, and health transitions, even though their usefulness in describing and understanding population and health trends in developing countries has been repeatedly called into question. The issue is particularly relevant for the study of population health patterns in Africa and sub-Saharan Africa, as the history and experience there differs substantially from that of Western Europe and North America, for which these concepts were originally developed.

**Objective:**

The aim of this study is two-fold: to review and clarify any distinction between the concepts of demographic transition, epidemiological transition and health transition and to identify summary indicators of population health to test how well these concepts apply in Africa.

**Results:**

Notwithstanding the characteristically diverse African context, Africa is a continent of uncertainties and emergencies where discontinuities and interruptions of health, disease, and mortality trends reflect the enduring fragility and instability of countries and the vulnerabilities of individuals and populations in the continent. Africa as a whole remains the furthest behind the world's regions in terms of health improvements and longevity, as do its sub-Saharan African regions and societies specifically. This study documents: 1) theoretically and empirically the similarities and differences between the demographic transition, epidemiological transition, and health transition; 2) simple summary indicators that can be used to evaluate their descriptive and predictive features; 3) marked disparities in the onset and pace of variations and divergent trends in health, disease, and mortality patterns as well as fertility and life expectancy trajectories among African countries and regions over the past 60 years; 4) the rapid decline in infant mortality and gains in life expectancy from the 1950s through the 1990s in a context of preponderant communicable diseases in all African countries; 5) the salient role of adult mortality, mostly ascribed to HIV/AIDS and co-morbidities, since the 1990s in reversing trends in mortality decline, its interruption of life expectancy improvements, and its reversal of gender differences in life expectancies disadvantaging women in several countries with the highest prevalence of HIV/AIDS; 6) the huge impact of wars in reversing the trends in under-five mortality decline in sub-Saharan countries in the 1990s and beyond. These assessments of these transition frameworks and these phenomena were not well documented to date for all five regions and 57 countries of Africa.

**Conclusion:**

Prevailing frameworks of demographic, epidemiological, and health transitions as descriptive and predictive models are incomplete or irrelevant for charting the population and health experiences and prospects of national populations in the African context.

Over the past six decades, life expectancy for the world's population increased from 47 years in 1950–1955 to 69 years in 2005–2010. By 2005–2010, life expectancy at birth in the most developed regions was 77 years, while it was 4 years shorter in Latin America and the Caribbean (73 years), 7 years shorter in Asia (70 years), 21 years shorter in Africa (56 years), and nearly 24 years shorter in sub-Saharan Africa (SSA) (53 years). These disparities are indicative of differences in the demographic and epidemiological changes that have taken place in various regions around the world. The increases in longevity have been accompanied by a historic shift in the cause-specific mortality risks in human populations, and researchers have developed a number of theoretical frameworks to describe and explain these patterns, including the demographic transition (1), the epidemiological transition (2–11) and the health transition (12–22). Over the past three decades or so, these frameworks have been used extensively by researchers from different disciplinary perspectives to study and compare the population and health experiences of various countries. These frameworks have been applied in studies of mortality, morbidity, health and development, population development, and health development in developed countries, in rapidly changing middle-income countries, and, to a lesser extent, in low-income countries (23–48).

For the vast majority of the past 140,000 years of human existence, birth and death rates were at very high levels – around 30–50 per 1,000 people. The transition to low birth and death rates was termed the ‘demographic transition’ by Notestein (1). This formulation offered a framework for describing and understanding population change, and during the 1960s through the 1980s it led to intense debates on the reasons for population growth and the means of population control (22, 45). The mortality component of this transition was first elaborated by Omran (2–11), who used the concept of the ‘epidemiological transition’ to describe and explain transformations in the patterns of disease occurrence and causes of death. By the 1990s, Omran's concept, which he had updated and elaborated over the years (2, 10, 11) had become a classic citation in demography, epidemiology, and public health, and it remains the most cited paper in population health (46). About that same time other authors had developed the concept of ‘health transition’ as either a revision or an extension of Omran's theory (12, 13, 29, 48) or as a new and distinct concept (19, 43, 49).

The extent to which these existing concepts provide an adequate framework for describing and understanding population health trends in developing in general and especially in Africa is unsettled (23, 28–45, 48–53) , particularly in light of three types of health problems facing that continent: 1) the HIV/AIDS epidemic, along with re-emerging infectious diseases such as tuberculosis; 2) the widespread presence of illnesses caused by common infectious diseases (notably malaria and childhood diseases) and malnutrition; and 3) the emerging epidemic of chronic diseases, accidents, and mental disorders. This study is a systematic review and critical assessment of the relevance of these frameworks for describing and understanding health, disease, and mortality patterns in Africa over the past 60 years, which is a sufficiently long period for gauging changes within the historical demography and epidemiology of Africa.

Hypotheses developed to explain the health, disease, and mortality processes as they occurred in the last three centuries in Western Europe and North America have been tested in other developed countries as well as in some middle- and low-income developing countries since the 20th century. However, Africa's historical epidemiology and demography have posed a challenge to researchers and policymakers because of the scarcity of data and evidence-based written sources in large parts of the continent in general and in SSA in particular (31, 33, 39–41, 54–61) . Health, disease and mortality processes have been taking place in African countries under various political, cultural, social, economic, demographic, structural transformations and institutional environments over the past 60 years and beyond.

The purpose of this study is two-fold. First, it reviews and clarifies any distinction between the concepts of demographic transition, epidemiological transition and health transition. When it comes to semantics in global health research, these concepts are often used synonymously in various contexts and from different disciplinary perspectives, without due caution to similarities and differences between them regarding their contours, their descriptive and explanatory dimensions, and their prognostic implications. Second, it identifies summary indicators of population health to test how well these concepts apply in Africa. There is no critical appraisal of these concepts in the context of all African regions and countries. As we will see, the relevant evidence points to the need for a new and different perspective for population and health changes in the African context. There are many examples between and within countries and over time where these transition frameworks obviously do not apply (62–70). Instead, the evidence indicates that the social, economic, political, cultural, and demographic contexts relate to the health, disease, and mortality patterns in Africa in ways that are quite different from the understanding derived from these perspectives.

## Study methodology

### Review on the demographic transition, epidemiological transition, and health transitions

For the review of the extant literature on the demographic transition, epidemiological transition, and health transitions, we searched OAJSE, Scopus, ScienceDirect, Scirus, PubMed, Google Scholar, SciCentral, MUSE, POPLINE, World Bank, World Health Organization (WHO), United Nations databases and publications for reviews and ascertained relevant publications from these and other sources, using subject headings or key words related to demographic transition; epidemiological transition; health transition; population change; population development; population health; health demography; public health in Africa; epidemiology in Africa; African demography; health, disease and mortality in Africa; cause of death in Africa; infectious disease; chronic disease; communicable disease; noncommunicable disease; SSA; Africa. Web pages of organizations active in areas relevant to this study, such as WHO, United Nations, UNICEF, FAO, and regional offices of organizations based in Africa working on or around health, disease and mortality in Africa, were also screened for further pertinent publications. Relevant materials from these searches are used in this study.

### Empirical data and methods of analysis

#### Data sources

For quantitative evidence spanning the last 60 years and involving all five regions and 57 countries forming Africa, data from the online latest estimates in the databases from the United Nations are used for assessing the levels, trends, and patterns in demographic changes as well as mortality statistics and characteristics in Africa from 1950 to 2010. We use the WHO mortality estimates by cause, age, and sex for the year 2008. WHO uses numerous data sources and epidemiological models to estimate the worldwide cause-of-death patterns. Such databases allow us to uncover patterns of demographic and epidemiological structures and processes and hypothesize their causal mechanisms and potential consequences on the health and disease patterns in Africa. National deviations from international patterns or those expected from theoretical perspectives will further enlighten our demographic and epidemiological understandings of the African epidemiological landscape and should pinpoint the specificities and similarities among and within countries given the social and cultural complexities of individual African countries. These databases provide a series of comparable national estimates from all African countries, and the completeness of their documentation reduces the risk of misuse. The reliability of these estimates for SSA in particular has been questioned (71). But they represent the best data available for all African countries since 1950. Although no sub-Saharan African country possesses reliable registration systems, model-based existing estimates of mortality statistics and characteristics for Africa indicate that SSA has the highest burden of disease in the world (64, 66, 72–74).

#### Methods of analysis

For each summary indicator selected for analysis, we will use a simple statistical approach for assessing changes over time. Let δ be the average yearly rate of change in indicators of interest between two consecutive years. Let µ_i_ be the proportion of events for a given year i. We have: µ_i + 1_=µ_i_ (1 + δ). To assess changes over time involving k (k=1, … n) time-dependent indicators, the equation is: µ_n_=µ_0_ (1 + δ)^n^, yielding δ=[(µ_n_ /µ_0_)^1/n^ - 1]. From this equation, the average yearly rate of change from say 1950 to 2010 is: µ_2010_=µ_1950_ (1 + δ)^60^. The yearly rate of decline needed to reach the targeted indicator level between 1950 and 2010, according to expectations from demographic, epidemiological and health transitions is readily available. For instance, the MDG4 is to reduce under-five mortality (U5MR) by two-thirds between 1990 and 2015; thus, the targeted U5MR for 2015 is µ_2015_=µ_1990_/3. The MDG5 is to reduce by three-quarters, between 1990 and 2015, the maternal mortality ratio (MMR); hence, the targeted MMR for 2015 is µ_2015_=µ_1990_/4. The yearly rate of decline needed to reach the targeted U5MR rates (respectively MMRs) from 2008 to 2015 set by MDG4 (respectively MDG5) is δ=[(µ_2015_ /µ_2008_)^1/7^ - 1]. The waiting times w from 2008, to reach the U5MR (respectively MMR) from 2008 to 2015 set by MDG4 (respectively MDG5), assuming the average yearly rate of change above is w=[log(µ_2015_ /µ_2008_)]/log(1 + δ).

## Perspectives of the demographic, epidemiological, and health transitions

The general approach to a transition framework is the characterization and explanation of a set of long-run irreversible changes of substantial social, ecological, cultural, political, behavioral or health significance with enduring global generalization, which are experienced as a society is transformed from one state or structure to another. The concept of transition has been used to describe dynamics involved in human history. It has been approached from different disciplinary perspectives. The transition concept was first proposed by demographers to describe the differing patterns of the components of population dynamics between and within countries with varying levels of socioeconomic transformation in the course of population development. Subsequently, the concept of transition has been used to describe historical trends in demographic, epidemiological and nutritional changes in human populations in the context of economic, sociocultural and political changes. It has been conventional wisdom in all earlier formulations to use three phases for the demographic transition (1, 75, 76), the epidemiological transition (2, 10, 11), the nutrition transition (77–81) and the health transition (12–21). These perspectives are often used synonymously, and it is warranted to clarify any distinction between them, in order to assess their relevance to concurrent changes and their implied health and disease patterns.

### Demographic transition

#### Definition

Demographic thinking in transition terms essentially began as early as 1929. It started as a description of demographic changes using a classification of populations into three groups according to different combinations of mortality and fertility levels resulting in three groups of countries with different population growth rates (75). Independently, Landry (76) described the demographic revolution characterized by three demographic regimes: primitive regime associated with subsistence economies constraining mortality reduction, intermediate regime with fertility decline due to late marriage and celibacy, and modern regime where fertility is an object of conscious limitation. The classic formulation of the demographic transition is ascribed to Frank W. Notestein (1). A fundamental concept in modern demography, the demographic transition is the intermingling description with explanation of the decline from high to low rates of mortality and fertility as historically experienced by populations in high-income societies of Europe, North America and Australia, and conjectured to be a universal principle expected to occur nowadays in populations of middle- and low-income countries. As a descriptive concept, the demographic transition is the characterization of the long-run situation of population change in three transitional regimes: a pre-transition regime with a quasi-equilibrium of high and fluctuating mortality and high fertility allowing a modest population growth or decline; a transition regime characterized by a transitory disequilibrium of declining mortality followed by declining fertility triggering population growth; and a post-transition regime with quasi-equilibrium of low mortality, low and conceivably fluctuating fertility and declining population growth rate. Rises followed by falls in the population growth rates result in the inverted U-shaped demographic transition. From an explanatory perspective, technological change and industrialization as the modernization progress unfolds and paves the way to the urban transition or the progression from low to high urbanization rates, are viewed as the crucible of demographic transition.

#### Description

The two key elements of the demographic transition are the mortality transition and the fertility transition from long-term quasi-equilibrium high levels to long-term quasi-equilibrium low levels: during the transition process, mortality is expected to decline first, followed by fertility decline with a time lag of 50 or more years between the mortality and fertility declines. The fertility decline is viewed as an adjustment made necessary by the decline in mortality in response to the forces of change. The transition begins with sustained declines in mortality, especially infant and child mortality. It is predicted that all societies will experience such a transitional or lag period during which birth rates exceed death rates by a substantial margin that will trigger rapid population growth. The underlying assumption of the demographic transition is that a population would move from one long-term quasi-equilibrium to another (1, 76, 82–86). During the pre-transitional stage (before 1800 in Europe), life expectancy at birth was less than 40 years with women bearing on average five to eight births. During the transitional stage (from 1800 to 1950 in Europe), life expectancy at birth was between 40 and 65 years with women bearing on average 2.6–4.9 births. In Europe where fertility decline followed mortality decline, the rate of natural increase (birth rates minus death rates) during the transitional period from 1800 to 1950 ranged between 0.5 and 1% per year. During the post-transitional stage (after the 1950s in Europe), life expectancy at birth exceeded 65 years with women bearing on average 2.5 or fewer births.

#### Explanation

There are two chief driving forces of the demographic transition. On the one hand, mortality decline is viewed as initiating the transition process and is generally considered as the most influential factor of fertility decline. Modern mortality decline was unprecedented in human history. This decline has been explained by the reduction of epidemics through vaccination and better hygiene, improved diagnosis and treatment of disease, reduction of famines, fewer deaths from violence and civil wars, reductions in infant and child mortality, and improved standards of living. On the other hand, the demographic transition amounts to the dual transition of mortality and fertility in response to forces of change including urbanization, economic development and cultural change, individualism, secularism, self-fulfillment, and changes in attitudes about and values related to family life. Certain cultural changes such as an increase in the value placed on investment in child human capital, may trigger both mortality and fertility declines, given the growing evidence pointing to the influential role of conditions in early childhood in determining adult health and human capital.

### Epidemiological transition

#### Definition

The epidemiological transition refers to the long-run shift in mortality and cause-of-death patterns inherent in the secular mortality decline from high to low levels embedded in a series of concurrent changes in population health. The concept was coined in the mid-1960s and published in 1971 in its first formulation by Abdel R. Omran (2). It provides an accurate characterization of the mortality transition from about 1750 through the 1950s ascribed mainly to the decline of infectious diseases, as experienced in Europe and North America. Omran's concept was initially influenced by theories of social evolution published by Riesman et al. (87) who divided social types into three historical stages based on Notestein's formulation of the demographic transition (1). By focusing on how concurrent changes affect the health of individuals within a population, a transition becomes a change in relationships between people and disease that occurs in a given time and space.

#### Description

The epidemiological transition is a descriptive and predictive model of change in health and disease patterns in human populations. It is formulated in five propositions embodied in: a three-stage three-model in its first formulation (2), a three-stage four-model in the update of its first formulation (10), and a five-stage six-model in its ultimate formulation (11) by the time of Omran's death in 1999.

The first proposition in all formulations of the epidemiological transition, like the demographic transition for which mortality decline and fertility decline are the two key elements of population change (1), relates to the relative role of mortality and fertility in the epidemiological transition: mortality is a central force within the complex dynamics of this transition, notably during its early phases where fertility decline in Western populations was gradual and occurred 50–75 years after mortality declined (2, 10, 11).

The second proposition in its first formulation states that ‘during the transition, a long-term shift occurs in mortality and disease patterns whereby pandemics of infection are gradually displaced by degenerative and man-made diseases as the chief form of morbidity and primary cause of death’ (2: 516). This second proposition is unquestionably at the heart of Omran's concept of the epidemiological transition, and has served as the framework of reference in most empirical studies and reviews. In keeping with the stage-wise approach inherent in the demographic transition, Omran's ultimate formulation of the epidemiological transition (11) entails five stages, in response to critiques from several authors (12–21, 23–49, 88–91) who have proposed extensions of the epidemiological transition stages, by proposing a fourth stage called age of delayed degenerative diseases or hybristic stage (88, 89) and a fifth stage (of emerging infectious diseases) (90, 91). There is also some analogy here that can be made with the literature on the demographic transition which has recently suggested additional stages of the standard demographic transition, the ‘second demographic transition’ (92) and the ‘third demographic transition’ (93).

Omran's latest formulation of the second proposition encompasses a set of distinct transitions: the changing patterns of disease and health (he termed ‘health transition’), the changing fertility and population age structure leading to ageing (he labeled ‘partially the demographic transition’), the changing lifestyles (he called ‘the lifestyle transition’), the changing health care patterns (he designated ‘the health care transition’), the medical and technological evolutions (he called ‘the technologic transition’), and the environmental and ecological changes (he termed ‘the ecological transition’) (11). Omran's earlier formulations of the epidemiological transition (2, 10) posited three evolutionary stages characteristically featuring all-cause mortality patterns and fertility patterns in the course of population change during the transition, and presumed that all societies were to go through these stages as did the now-developed countries of Western Europe and North America, just as conjectured in the demographic transition (1). Omran's ultimate formulation is modified to contrast the five successive stages in western societies with the three successive stages in non-western societies (11). By drawing such distinction between western and non-western societies, Omran has attempted to address some of the various assessments of the epidemiological transition since its inception (12–22, 43–50, 88–91) . The distinction is based on mortality and fertility patterns, cause of death structure, health care, and socio-economic contexts. Mortality decline in non-western countries did not occur until the middle decades of the 20th century when the speed of such decline was unprecedented in comparison to the previous experience in the West. Mortality by cause of death in non-western countries was projected to shift in predominance from communicable diseases dropping from being responsible for 42.1% of all deaths in the less developed regions in 1970 to 19.4% in 2015, while neoplasm and circulatory disorders will rise from accounting for 21.6% of all deaths in 1970 to 48.9% in 2015, in tandem with life expectancy at birth which will rise from 57.5 to 68.5 years (11).

The first stage (age of pestilence and famine), is characterized by high and fluctuating mortality rates, variable life expectancy with low average life span of 20–40 years, and periods of population growth that are not sustained. The disease patterns that emerged are determined by increasing microbial exposures, dietary deficiencies, illnesses due to inadequate food storage, communicable and endemic diseases over wide geographic regions and increased mortality. In western societies and depending on the country, it entails the period from the Stone Age (or the extension of medieval or pre-industrial disease patterns) to the late 18th or early 19th centuries and was characterized by high and fluctuating mortality just as fertility was high, leading to slow and cyclic population growth. The average life expectancy at birth was short and variably swinging between 20 and 30 years and life expectancy for males was higher than or equal that of males, infant mortality exceeded 200 deaths per 1,000 live births, MMR was high (exceeding 1,000 per 100,000 live births), and infectious diseases, malnutrition and maternal complications claimed up to 75% of deaths while heart diseases and cancer claimed 6% of deaths. In non-western societies, the first stage is the longest length spanning from the medieval times to the middle decades of the 20th century: mortality is extremely high and fluctuating with peaks during the years of epidemics, famine, crop failures and wars, thus precluding sustained population growth; infant mortality exceeds 200–250 deaths per 1,000 live births, MMR exceeds 1,000–1,200 deaths per 100,000 live births, and life expectancy is short and oscillates between 20 and 35 years; disease pattern features a distinct predominance of communicable, maternal, perinatal and nutritional diseases; and fertility is extremely high (7–10 births per woman) and childbearing starts at a young age and runs through the entire reproductive span.

The second stage (age of receding pandemics) is marked by a steeper decline in mortality as epidemics occur less frequently, and an increase in average life expectancy from around 40 to 55 years of age. This transition phase is characterized by a shift in patterns of disease and mortality from primarily infectious diseases to chronic and degenerative diseases. This shift is supposed to be accompanied by a shift in the population age distribution as early infectious disease deaths decline and deaths from chronic and degenerative disease increase. In western societies, this stage covered the period from the mid-18th century to approximately 1914. It saw the disappearance of the major acute infections as causes of death. The average life expectancy at birth increases steadily from about 30 to 50 years while infant mortality declines gradually to below 200 per 1,000 live births. Fertility remains at high levels and the widening gap between birth and death rates results in rapid population growth as well as a young age structure. During the latter part of this stage and usually after a time lag of 50 or more years from the onset of mortality decline, fertility decline takes place. In non-western societies, this second stage ended in the 1960s through to the 1990s and its beginning was delayed in some non-western countries until the 1940s and 1950s, when the onset of mortality decline occurred with the start of the recession of epidemics.

The third stage (age of degenerative, stress and man-made diseases in western countries; or age of triple health burden in non-western countries) features infectious disease pandemics that are replaced as major causes of death by degenerative diseases, with infectious agents as the major contributor to morbidity and mortality overtaken by anthropogenic causes. With declines in mortality rates, average life expectancy increases to exceed 70 years, fertility becomes more important to population growth, and the anthropogenic and biologic determinants of disease also change. In western societies, it ran from the second half of the 19th century to the late 1960s. This stage is a manifestation of the increasing prevalence of man-made diseases (e.g. radiation injury, occupational and health care hazards, chemical and biological warfare, environmental pollutants, motor vehicle and aviation accidents, carcinogens in the environment and in industry and food additives), stress-related diseases (e.g. depression and other mental illness, violence and drug dependency) as well as heart diseases, cerebrovascular accidents (strokes), cancer at various sites, diabetes, chronic obstructive pulmonary disease and metabolic disorders. These diseases have been progressively replacing infectious diseases and gross malnutrition which continue to be among the leading causes of morbidity and mortality without being the top causes of death. Mortality continues to decline and the average life expectancy at birth rises gradually from about 50 to 75 years or more, triggering population aging. It is during this stage that fertility becomes a crucial factor in population growth. In non-western societies, this third stage is termed the age of triple health burden: 1) unfinished old health problems including communicable diseases, perinatal and maternal morbidity and mortality, malnutrition, poor sanitation, rampant poverty, low literacy, overpopulation, limited access to health care and clean water; 2) rising new set of health problems including degenerative diseases (heart diseases, stroke, cancer and metabolic disorders), stress and depression, and man-made diseases as in western countries; and 3) lagging or ill-prepared health systems and medical training for the triple demands of quality services consisting of dealing with acute diseases generally resulting in short-term care, initiating prevention and care for chronic and non-communicable diseases (NCDs) usually requiring long-term medical or rehabilitation care, and handling problems of ageing. It started in the 1970s or later and its takeoff varies across countries given its transition model. During this stage, fluctuations more or less disappear and mortality continues to decline and eventually approaches stability at a relatively low level. The average life expectancy at birth rises gradually until it exceeds 70 years.

Omran (11:107) did not consider additional stages of the epidemiological transition for non-western countries, arguing that ‘it is quite unlikely that these countries will enter the fourth stage in a manner similar to the one experienced by western countries’. Omran (11) noted six distinctive features of non-western transition. First, throughout the non-western countries, poverty, limited education, low status of women and slow pace of development have been major obstacles to well-timed and successful takeoff. Second, the timing of transition is crucial for the differentiation of the non-western transition. Third, since mortality decline was delayed until well into the 20th century, it was forcibly influenced by the availability of new health discoveries such as chemotherapy, antibiotics, insecticides, food quality and sanitary measures. Fourth, aging in non-western societies was delayed, but increased at a relatively fast rate in the later decades of the 20th century. Fifth, fertility decline in non-western countries was harder to initiate and required organized family planning campaigns to promote smaller family size norms and rise the age at marriage. Finally, the overlap of stages was much wider in the non-western transitions which Omran (11) classified further into three transition models.

The fourth stage is the age of declining cardiovascular mortality, aging, lifestyles modification, emerging and resurgent diseases. This new stage draws on later contributions (88–91). It begins with a plateau in epidemiological history where mortality reached at once equilibrium with a life expectancy at birth in the 1970s at a value which was then believed to be close to the biological limit to the average length of human life. Olshansky and Ault (88) noted that a few years prior to Omran's seminal publication (2), the United States and other Western countries began to experience a rapid decline in cardiovascular mortality which occurred around 1970 in many developed countries; they proposed this cardiovascular revolution as a fourth stage of the transition labeled ‘age of delayed degenerative diseases’. Characteristically, the age pattern of mortality by cause of death is unaltered from the third stage to this fourth stage, but the age distribution of deaths from degenerative causes shifts progressively to advanced ages with associated increases in the size of the population at advanced ages and on the health of the elderly especially the oldest old. There are further increases in life expectancy approaching 80 to 85 years or longer notably for women, increased disease chronicity, and aging accompanied by rising medical costs for individuals and the state.

The fifth stage is the age of aspired quality of life, equity, development and social justice for all. This stage is a futuristic stage with paradoxical longevity, chronic morbidity, disabling impairments, isolation and decline in social status, loss of independence, new morbidity, mounting medical, long-term care and nursing costs, persistent inequities, inadequacies and disparities between people, communities and countries because of the polarization of socioeconomic status within and between countries. At this stage of the epidemiological transition, much increased chronicity will be a common feature of degenerative, stress and man-made diseases which will be higher than the causes of morbidity, disability and mortality. Omran (11) conjectured that tobacco will be the most villainous disease risk factor in this stage.

#### Explanation

The following three propositions explain the differentials in epidemiological transition.

The third proposition is that there are age and sex differentials that come with the epidemiological transition. Regarding age differentials, the transition usually favors the young over the old: the most significant impact of the transition is the fall of infant and child mortality and maternal mortality and the rise of mortality at 50 years or older (10). Childhood survival gradually improved as pandemics receded in response to better living standards, improved nutrition and sanitation and modern public health measures in Europe. This was also apparent for the United States between 1880 and 1970, and for England, Wales and Chile (2, 10).

The fourth proposition is that the shifts in health and disease patterns that characterized the epidemiological transition prior to the 20th century have a closer association with rising standards of living and improved nutrition than with medical progress. In contrast, the 20th century transitions prevailing in the less developed countries are initiated by medical progress, organized health care, and disease control programs that are usually internationally assisted and financed, and thus largely independent of the socioeconomic level of the country. Further maturation of the transition, however, depends on a beneficial synergy of health care progress and socio-economic development. Likewise, the fertility transition prior to the 20th century was largely determined by social and economic progress in countries that were more or less familiar with traditional methods of fertility control. These methods, as well as rising age at first marriage, were the major intermediate variables for fertility reduction. The more recent fertility decline in some developing countries, on the other hand, has been dependent on organized efforts of family planning in conjunction with socio-economic development.

The fifth proposition is that distinctive variations in the pattern, pace, determinants, and consequences of health, survival and population changes differentiate six transitional models of the epidemiological transition: the classical or Western transitional model (e.g. Western Europe, North America, Japan, and Australia), the semi-western/accelerated model (e.g. Argentina, Paraguay), the non-western rapid transition model (e.g. Chile, Costa Rica, Cuba, Jamaica, Martinique), the non-western upper intermediate transition model (e.g. Brazil, Mexico), the non-western lower intermediate transition model (e.g. Paraguay, Peru), and the non-western slow transition model (e.g. Bolivia, Haiti, Nicaragua) (2, 10, 11).

The western transition model describes the transition in western societies which have entered the fourth stage of the transition during the past three centuries. It is characterized by the shift of birth rates from 30 to 35 per 1,000 population to less than 15 per 1,000 population, the shift of death rates from 30 to 35 per 1,000 population to less than 10 per 1,000 population, the shift of short life expectancy of 30–40 years to unprecedented long life expectancy of 80–90 years and more. Mortality declined gradually and was due primarily to social, economic and environmental improvements, better nutrition and personal health habits. During the transition, pandemics and major epidemics receded and were gradually but not totally replaced by degenerative, stress-related and man-made diseases; these changes in disease patterns were associated with improvement in life expectancy notably for children, young adults, and females of reproductive age. Fertility decline in this Western model was also gradual and occurred 50–75 years after mortality declined, but this sequence of fertility decline following mortality decline was not universal because in France and some European parishes, fertility and mortality declines almost coincided.

The semi-western/accelerated model describes the experience in eastern Europe, the former USSR and Japan, and in European populations living outside Europe, North America or Australia at various historical epochs (e.g. Europeans living in Argentina, Israel, South Africa). In most of these countries, a slow process of modernization had begun prior to the drop in mortality in the 20th century, which was determined by general social improvements as well as by sanitary and medical advances. Wide use of abortion typically helped accelerate the fertility transition, like in Japan (10). By and large, countries in this model have not entered the fourth stage of the epidemiological transition, many of these countries exhibit a continuing rise in cardiovascular mortality, and some members of the former USSR have experienced lost years of life expectancy due to social and economic crises.

The other transition models apply to non-western countries, describing epidemiological changes over time in less developed countries where mortality decline took place no earlier than the 1930s through the 1950s and fertility decline was delayed further until after 1950. The sharp decline in mortality in tandem with high fertility led to unprecedented rates of population growth during this period, and Omran (11) distinguished these countries not only in terms of differences in the post-war patterns of mortality and life expectancy, but mostly given the speed, timing and magnitude of change in fertility decline.

The non-western rapid transition model describes the experience of rapidly industrializing or socially developing countries and territorial islands (e.g. Taiwan, Hong Kong, Singapore, South Korea, Sri Lanka, Mauritius, China, Chile, Cuba, Barbados, Costa Rica, and the French overseas departments such as Reunion). The onset of mortality decline to moderate levels in these countries was one or more decades before 1950 while fertility decline to less than five children per woman was delayed until sometime in the 1960s. These countries are still in the third stage of the epidemiological transition, but have been experienced also the triple disease burden.

The non-western (upper and lower) intermediate transition model embodies the experience middle- or low-income countries whose changes in mortality and disease patterns and fertility patterns are situated between the rapid and slow transition models. Countries in the upper non-western intermediate transition model include Indonesia, Colombia, Mexico, Brazil, Panama, Venezuela, Tunisia, Lebanon, and Thailand. Countries in the lower non-western intermediate transition model comprise India, Egypt, Morocco, Ecuador, Peru, Paraguay, and the Dominican Republic. These countries still face a dual burden of continuing communicable diseases and malnutrition and the rising prevalence of NCDs, often in combination with the emerging diseases such as HIV/AIDS or the resurgent diseases such as malaria.

The non-western slow transition model describes the experience of the least developed countries and some of the less developed countries in Latin America, Asia and Africa. In these countries, mortality started declining to moderate levels after 1950 while fertility remained at high levels until the 1990s. These countries also face the dual burden of the continuing preponderance of communicable diseases and malnutrition and of the rise in degenerative and man-made diseases, along with HIV/AIDS, malaria, tuberculosis, and other emerging or resurgent diseases; they also have been ill-prepared to handle any one health problem.

### Health transition

#### Definition

The health transition refers to the various components in combination in a series of concurrent changes in population health during the development, and their implications for health and social policies and programs. For instance, the functional component represents change in functional health status (i.e. abilities and disabilities) of the population and the gerontological component denotes the increasing proportion of people at old and very old ages with their connected health problems. The health transition is the latest conceptual effort made by social and public health scientists to describe and explain secular transitions in population and health dynamics. However, as we show below, its conceptual clarity and contributions, empirical foundations and prognostic implications both regarding historical and contemporary populations, remain typically blurred and unmapped.

#### Description

Several appraisals of the epidemiological transition have suggested revisions to its earlier formulations (12, 13, 23, 26–32) or have rejected it altogether as not relevant to health and disease patterns of the low- and middle-income countries (18, 19–22) . Undeniably the most successful attempt of such revisionism is the concept of the ‘health transition’ meant to be a wider framework that included both population change, health system, epidemiological profile of the population and ways in which societies respond (or not) to changing health situations as a result of cultural, social, and behavioral determinants (12–21). The ‘health transition’ gained considerable traction in the 1990s. While the health transition perspective seeks to provide some causation to the explanation of the secular changes in health and disease patterns in human populations, health transition represents different conceptual entities depending on the school of thought which have made progress towards a coherently crafted framework beyond Omran's epidemiological transition a daunting endeavor. The Caldwell's school of thought views health transition as rooted in the cultural, social and behavioral determinants of health (18–21, 49), while scholars associated with the Frenk's school of thought (12, 13, 23, 26–29) view it as a revision or an extension of the Omran's epidemiological transition (2, 10).

Caldwell's perspective on health transition (19) departs from Omran's epidemiological transition (2, 10, 11) and Frenk's elements of health transition (12, 13). It builds instead on a paper by Simons (94) published in a volume edited by Caldwell and Santow (14) around the same time as the work of Frenk and colleagues (12). Caldwell's school of thought considers the health transition and improvement in health in circumstances of poor economic growth typical of developing countries such as those of SSA. What Caldwell and Santow (14) refer to as ‘the health transition factor’ is very influential in this perspective: societies with similar levels of income and provision of health services can exhibit very different levels of health and mortality. This can also apply to different cultures, families or households even within the same societies, indicating a behavioral or attitudinal effect at work that has to do with lifestyles. The main argument embodied in Caldwell's view of the health transition is that the driving forces of population and health changes in different epochs are cultural, social, and behavioral in nature in both western and non-western societies (18, 19, 20, 21) . The evidence from changes in non-western societies suggests that a considerable part of the gap in causation is explained by individual and social change. At the individual level, new attitudes and behavioral patterns took advantage of socioeconomic opportunity structures and medical advances to improve survival chances. First, death is abhorrent and must be avoided at all costs, and there must be an absolute commitment to ensuring the survival of all members of the community. Second, there must be a feeling in all individuals of a sense of active individual commitment to participating in ensuring survival of each other by avoiding death through the reduction of the risk of harm or death, rather than believing that external forces, resources, physical or moral entities will do it for individuals. The most powerful instrument that has been shown to accelerate those aspects of social change that enhance health improvement and survival has been found to be raising individual consciousness and sense of responsibility, particularly through formal education in general and female education notably (14, 15, 19). The routes to low mortality in such resource-disadvantaged settings is the establishment of a democratic health system, by implementing a network of functional health facilities that are reachable by the local populations with emphasis on accessibility (free or cheap) of the poor of rural and urban areas, as well as universal health insurance coverage of some form to them. Perhaps Caldwell's perspective on health transition is best styled in his following words:

… During the long contact with the important institution now known as the International Centre for Diarrhoeal Disease Research, Bangladesh (ICDDR,B), I have been astonished at the lack of interest in identifying necessary behavioural changes, and trying to supplement them. Most of the ICDDR, B's resources in the diarrhoeal area have been employed to identify diarrhoeal pathogens, and to search for methods of immunization… Except in the area of inducing parents to make and employ oral rehydration solutions, however, practically nothing on studying, or inducing individual or societal hygienic behavioural change has ever been attempted. The focus has always been on pathogens and therapies rather than people. (19: 125).

Frenk's views of the health transition were articulated in two publications in the late 1980s and early 1990s (12, 13). Frenk et al. (13) propose that the concept of epidemiological transition be replaced by the wider concept of health transition, which would include not only the development of epidemiological characteristics within the overall health situation, but also the ways in which societies respond to the health situation and vice versa. This perspective considers the health transition in the context of middle-income countries facing new challenges for health care, in order to account for response of the organized health system to long-term changes in the health conditions of populations. More recently, Vallin and Meslé (29) have attempted to broaden the scope of this concept to represent situations of convergence and divergence in mortality both in developed and developing countries. Frenk et al. (12) propose some corrections and additions to the original three-stage-three-model version of the epidemiological transition (2). In criticizing Omran's formulation, Frenk et al. (12) argue that between the countries in the advanced stage of the epidemiological transition and those in the initial stage lies a third group that is undergoing a new transition experience quite different from that of the developed nations. They describe such countries as belonging to the ‘protracted and polarized’ transition model. This model has four characteristic features. First, the transition stages are overlapping: the stages of the transition do not follow a sequential order but exhibit considerable overlap. Second, there are counter-transitions: the shift from high mortality to high morbidity occurs not only for degenerative diseases but also for infectious diseases as in some developing countries (e.g. case for HIV/AIDS where morbidity rates are increasingly higher than mortality rates as treatment become available to a greater proportion of the population). Third, the transition is protracted: the transition process is not clearly resolved as countries exhibit both infectious and chronic diseases. Fourth, there is an epidemiological polarization: a polarization is seen to occur between population subgroups with the poor and rural populations succumbing to the pre-transitional pathology while the urban populations experience post-transitional pathology. Frenk and colleagues (12, 13) illustrate this perspective with evidence from the epidemiological transition in Mexico quite different from the one experienced in western and North American countries: in the 1950s, the 10 main causes of death already included many chronic and degenerative diseases such as heart conditions, tumors and cerebrovascular problems; infectious diseases caused more than 30% of all deaths. This is seen as the first description of the protracted epidemiological transition. The epidemiological polarization in Mexico is largely explained by the different pace of change in the causes of ill-health among the social classes.

Vallin and Meslé (29) suggest that health transition be characterized as an extension of the epidemiological transition. Within the concept of health transition tied to the healthcare system as proposed by Frenk (12, 13), Vallin and Meslé (29) consider the full process in term of divergence/convergence sequences inferred by successive major changes in health technologies and strategies. The entire health transition process thus breaks down into successive stages, each including a specific divergence-convergence sub-process. The first stage of the health transition is Omran's three-stage epidemiological transition in its classic formulation. The second stage of the health transition is the cardiovascular revolution. There is now overwhelming evidence indicating that Omran's third age of the epidemiological transition, the age of degenerative and man-made diseases (2, 10), was not the final one as also acknowledged by Omran (11). The third stage is the ‘slowing the ageing process’. This stage relates to the most recent trends in life expectancy for Western industrialized countries. The conjecture hinges on the view that the new approaches toward the elderly developed since the 1980s may be creating a new stage of health transition, which a handful of countries may have already experienced. The impact of the decline in cardiovascular mortality mainly comes from the oldest age groups. In light of this increasing role of the oldest ages in the decline of cardiovascular mortality, increasing advances in the fight against these diseases are tied up with more general progress against ageing. The current cause-of-death classification is certainly not sufficiently ageing-related to allow a pinpointing of the impact of current strategies on such ageing process.

#### Measuring health and describing the course of health transition

Given the intricacies of measuring individual and population health over time and across cultures, Johanssson (95) recently proposed three strategies for measuring health in an attempt to reach conclusions about the course of the health transition and how such concept may be related to historical and contemporary trends in mortality. The first strategy is to continue the use of mortality data to assess health trends, as most researchers have been doing (12, 13, 23–29, 33, 34), assuming that rises in life expectancy signify improvements in health. This strategy is tantamount to suggesting that rising life expectancy is all that matters, so that comparing life expectancy levels between countries during their economic development can measure health inequalities. The second strategy rests on a medicalized life course perspective on health and entails using data that are relatively culture-free (e.g. blood pressure) to objectively determine individual health by measuring its diminishing from healthy state through sickness and disability; hence, the health transition becomes a ‘doctor's transition’, based on comparable age cohorts at different points in time during the rise of life expectancy. The third strategy is to measure the health transition in all its complexity in its multifaceted, multilevel and life-course dimensions relevant to health policy. These second and third strategies are not readily feasible and require a formidable amount of cooperation across disciplines, large-scale resources and skills which are not commonplace.

### Comparing and contrasting the demographic, epidemiological and health transitions

#### Temporal coincidences between the demographic, epidemiological and health transitions

Four distinct historical periods have marked the mortality transition in the modern world described in the demographic transition (96) and which were used in subsequent efforts to describe and explain secular transitions in the epidemiological and health transitions. The first period runs through the first half of the 19th century. In Western Europe, mortality reduction is most clearly identified in the latter part of the 18th and first half of the 19th century. The second period covers the last third of the nineteenth century up to World War I. During this period, there was a revolution in medicine induced notably by the discoveries of Pasteur and Koch. The resulting reductions in child mortality and subsequently in infant mortality were responsible for much of the mortality decline particularly in mortality from diseases such as diarrhea and tuberculosis. During the inter-war period, sustained gains were achieved especially in medicine and health education, instilled by progress made during and after World War I. The third period involves the years during World War II and the following period through the 1960s. During this period, there was an explosion in the use of antibiotics, initiated by Fleming's discovery of penicillin and its synthesis in 1943. The cumulative effect of these developments has been a dramatic reduction in epidemic and communicable diseases. The fourth period covers the period since the 1970s when important gains have been achieved in reducing mortality from cardiovascular diseases, and particularly in increasing the longevity of older adults. The adoption of modern lifestyles has not been responsible for increased mortality from degenerative diseases until recently, because of the high incidence of communicable diseases.

#### Similarities

A common feature of these three transition frameworks of investigation of changes involves a progression through a sequence of three stages in their first formulations. The idea of convergence is a general basis of these frameworks, not only for mortality and life expectancy trends but also for fertility (97). This notion of progression through the stages of transition is the subject of a substantial and ongoing debate; such stage-wise linear approach to change is a major shortcoming of these concepts. To the extent that mortality data track the health status of a population at all ages, the mortality transition may proxy any separate health transition (96), provided that more specialized measures of mortality are produced to highlight the importance of various components of concurrent changes in the production of health during development.

Frank Notestein's formulation of the demographic transition (1) is by and large the most influential framework since the 1950s. The epidemiological and health transition are tied to the demographic transition in at least two respects (the notions of transition stages and mortality transition), but also represent distinct constructs in their own right. The Notestein's approach to mortality analysis is most fully elaborated in Omran's epidemiology of the population change (2, 10, 11) recognizing that mortality transition involves more than simple quantitative reductions in mortality levels and their short-term fluctuations. Similarly, the concept of health transition is closely related to sustained progress at reducing mortality among infants and adults and to improvements in life expectancy by changing the patterns of disease and cause of death at older ages in the context of well-equipped and functioning healthcare system. The mortality transition refers to the secular decline in mortality and its associated changes in mortality patterns of age and cause of death. The epidemiological transition includes the mortality transition and additionally embraces a broader set of changes in morbidity and the disease environment. The health transition in Caldwell's sense includes the social and behavioral changes that parallel the epidemiological transition and may do much to propel it (16). The concept of health transition proposed by Frenk et al. (12, 13) is tied to the healthcare system.

#### Differences

The main aspect that separates the epidemiological transition (2, 10, 11) from the demographic transition (1) is the addition of a new element – a shift in cause-of-death patterns and the stage-wise characterization of the transition stages by the configurations of the causes of death as well as the influences on them. Despite his reliance on its main concepts, Omran explicitly rejected the demographic transition as a theoretical framework, and formulated the epidemiological transition in an attempt to provide a comprehensive approach to the dynamics of the mortality–fertility transition (4). In his view, the demographic transition ignored too many variables and too many historical developments that did not fit the theory (e.g. the postwar baby boom), as well as assuming a single path of population development based on socio-economic progress. Omran consistently emphasized the importance of motivation rather than economic determinants in explaining why birth control was embraced enthusiastically in some places and rejected in others (2). He further believed that motivation could be promoted through health programs, directly by explaining the health benefits of family planning and indirectly by decreasing infant mortality and thus reducing the need for large families. For Omran (2, 10, 11), the key difference between epidemiological transition and demographic transition is that the former unlike the latter allows for multiple pathways to a low-mortality/low-fertility population regime.

A feature of the concept of the health transition that situates it apart from the demographic and the epidemiological transitions is the idea that there is more to health than death, especially at older ages around the world. For instance, it is known that the health of the elderly can deteriorate even if life expectancy is stable or rising at older ages. Continuing improvements in high life expectancies have made it imperious to develop measures of population health such as active life expectancy (ALE), disability-adjusted life expectancy (DALY) and quality-adjusted life years (QALY) (98) which are apt to track the health status of a population without relying on mortality data alone. However, since health research has generally relied on quantitative data and analysis, Johansson (95) anticipates that researchers will continue to use mortality data as a proxy for health trends and to treat the health transition just as another name for the mortality transition.

## Changing contexts of health, disease and mortality patterns in Africa

### Brief history and experience of Africa

Information on the situation in Africa before the independence years in the 1960s is sketchy and at best conjectural. Kuczynski's study on the Cameroons and Togoland, published in 1939, was the first critical investigation of information on the population of Africa and was largely negative concerning the possibility of substantial knowledge on the basis of the information then available (99). By the 1960s, conditions were relatively favorable (100), but by the end of the 1970s there were clear signs that the post-colonial state was not only falling short of its ambitious designs, but facing a systematic crisis, and Africa was unable to point to any significant growth rate or satisfactory index of general well-being (101).

The 1980s were a decade of growing distress for much of Africa, and since the 1980s, while other parts of the world have significantly prospered, African economies have experienced numerous disruptions. For sub-Saharan countries, there was an average negative growth rate of 2.8% from 1980 to 1987 (102). All African countries but Botswana and Mauritius were overwhelmed by unsustainable debts, facing negative trends in many primary commodity markets. Shunned by much of international capital, African states had little option but to accept, at least formally, the structural adjustment programs proposed by the international financial institutions backed by the donor community as the condition for continuing assistance in the late 1980s and beyond (103). The budget reductions required by structural adjustment programs compelled cutbacks in social expenditures. These trends substantially weakened states for which studies already showed that educational and health expenditures had less success in improving health and educational attainment than comparable outlays in Asia and Latin America (104).

While at the beginning of the 1980s the external diagnosis of an emerging African crisis was essentially economic, by the end of the decade the exegesis of the post-colonial state condition led to a more far-reaching conclusion: the malady afflicting Africa was not simply economic but more fundamentally political and required democratic reforms. The kinds of political and economic arrangements that characterized the post-colonial state had become fundamentally unviable in a changing global environment.

The 1990s and beyond represented a fundamentally new era in African politics, with the rise of multiparty systems. A vision of a new type of Africa state was offered by the New Partnership for Africa's Development (NEPAD), which pledged an economically reformed, politically democratic, and governmentally accountable and transparent Africa. However, the resulting partially reformed states proved to be substantially weakened, and new patterns of civil conflict and internal warfare appeared in a number of countries. Governments were overthrown by peripheral insurgents eight times in the 1990s: Chad (1990), Liberia (1990), Ethiopia (1991), Somalia (1991), Rwanda (1994), and Sierra Leone and both Congo and the Democratic Republic of Congo (1997). The spread of civil conflict in Africa in the 1990s stood as a metaphor for weakened and unstable nations. More than a quarter of African states experienced armed internal conflict during the decade, and another quarter faced prolonged political crises and turbulence (105), some of which are on-going.

### Conflict and instability

Over the last 50–60 years, African countries have experienced a variety of social, economic, and political crises that have affected population health and human longevity.

Politically, the 1950s and 1960s signaled the beginning of a new era for most of Africa (106–108), as the progress towards an orderly and peaceful transition of power from the colonial powers to the indigenous people began. Today, Africa contains 57 sovereign countries, most of which still have the borders drawn during the era of European colonialism. During the period from the early 1960s to the late 1980s, Africa had more than 70 coups and 13 presidential assassinations (109). Overall, 34 out of 52 African countries have experienced political instability (PI) as measured in 1996 or 2009, and 23 countries have witnessed a deteriorating political situation between 1996 and 2009. Since the 1960s, several African countries have experienced political coups, ethnic violence, oppressive dictators, and the region has endured many conflicts leading to humanitarian crises and mass migration of refugees.

The most devastating military conflict in modern independent Africa has been the Second Congo War, which by 2008 has claimed the lives of an estimated 5.4 million people. Since the 1960s, many western African countries have also been submerged under PI, with notable civil wars in Nigeria, Sierra Leone, Liberia, and Côte d'Ivoire, and a succession of military coups in Ghana and Burkina Faso. Southern Africa is distinct from the rest of Africa in that it has been exempt from wars.

Despite democratization trends since 1990, PI has been widespread in Africa. From 1956 to 2001, there have been 80 successful coups d’état, 108 failed coups d’état, and 139 reputed coup complots in 48 sub-Saharan African independent states (110). Elite PI remains widespread in SSA, in contrast to other regions of the developing world. Military-led PI has been shown to have deleterious effects on economic growth and human development in SSA and is a major cause of the enduring food, economic, and health crises in many African countries. This African context has huge implications on the capacity of the continent to set a path of continuous and sustainable social environment for human health development; unless Africa and Africans consciously change the course, their future may resemble their past.

### Disease

Although countries in Africa share certain similarities, such as conflict-prone environments, they differ greatly in many respects, including demography, epidemiology, culture, history, politics, and education. As such, it is not surprising to find that HIV epidemics across Africa range from highly concentrated to highly generalized, and the responses by African nations have been equally varied, ranging from intense interventions to complete denial of HIV disease.

Saying that HIV/AIDS has had a large impact in SSA is a gross understatement. Sixty-eight percent of people living with HIV worldwide live on the African continent, where every year 76% of all AIDS-related deaths in the world occur. In 2007 Africa accounted for 68% of new HIV infections in the world. At the same time, trends in the prevalence of HIV among adults aged 15–49 years shows great heterogeneity across countries. In 1990 over 10% of the adult population was HIV-positive in three countries (Zambia, Zimbabwe and Uganda); by 2000 the prevalence had risen to 24.8% in Zimbabwe and 14.4% in Zambia but had declined to 7.3% in Uganda. Botswana (26% and 24.8% in 2000 and 2009, respectively) and Lesotho (24.5 and 23.6% in 2000 and 2009, respectively) are the two countries with the speediest increase in HIV prevalence, having grown from 0.8% in Lesotho and 3.5% in Botswana in 1990.

Thanks to better HIV/AIDS treatments using antiretroviral therapies (ART), morbidity and mortality have decreased in HIV patients with advanced disease. However, as the number of patients on antiretrovirals increases, more and more people are living longer with HIV, raising new challenges. Furthermore, the majority of African countries have ART coverage under 45%, meaning that less than half of Africans who need antiretroviral therapy actually receive appropriate care, and current treatment guidelines often delay initiation of antiretroviral therapy during the early stages of disease.

The future of the epidemiological landscape of African countries will greatly depend on strategies to build capacity for prevention, treatment, and care of HIV/AIDS in Africa. If African states are to assume greater responsibility for responding to the HIV/AIDS epidemics, a major requirement will be to strengthen health-care systems in the region by building institutional and human resource capacity (111, 112).

Between 1990 and 2010, the prevalence of tuberculosis has increased in 25 African countries, and Africa is facing the worst tuberculosis epidemic since the advent of the antibiotic era (113). Driven by the HIV epidemic and compounded by weak health-care systems, inadequate laboratories, and conditions that promote transmission of infection, this devastating situation has steadily worsened, exacerbated by the emergence of drug-resistant strains of tuberculosis. The WHO estimates that the average incidence of tuberculosis in African countries more than doubled between 1990 and 2005, from 149 to 343 per 100,000 population – a stark contrast to the stable or declining rates in all other regions during this period (114). Although HIV is Africa's leading cause of death, tuberculosis is the most common coexisting condition in people who die from AIDS.

### Economic factors

In 2009, gross domestic product (GDP) per capita – a proxy for economic development – ranged from $97$ in the Democratic Republic of Congo to $8,011 in Equatorial Guinea. Although it has abundant natural resources, Africa remains the world's poorest and most underdeveloped continent. The informal sector is the largest contributor to GDP in the regions where agriculture is predominant, such as in SSA. Agriculture has always been and remains the dominant activity for the majority of Africans. Most agricultural activity is subsistence farming, which has made agricultural activity vulnerable to climate change and global warming. SSA is the region of the world with the largest estimates for the contribution of informal sector to GDP: nearly two-thirds including agriculture and one-third excluding agriculture.

Although over 50% of the adult population is employed in all African countries, labor force participation is dominated by the informal employment (115–119). The informal sector represents 80.4% of total employment in SSA, where 88.6% of women are more likely to be self-employed in the informal sector (118). This affects health because informal employment is usually characterized by an absence of social protection, in particular, health coverage (115, 118), which leaves large majorities of the continent's populations especially vulnerable to disease and other health issues.

### Health-care systems

In the absence of vaccination, measles is estimated to infect virtually the entire population with the exception of isolated communities (120), and measles vaccination rates are sensitive indicators of functional public health systems (121, 122). The joint WHO/UNICEF (123) estimates indicate that measles-containing vaccine (MCV) coverage rates were lowest for the African region.

Vaccine rates indicate a great diversity in the functioning of the public health systems across African countries. In 2010 estimated coverage of MCV rates ranged from 46 to 94%. There were 12 African countries with MCV coverage of 65% or lower, indicative of the least functional public health system, while coverage was at least 90% in seven countries. The regional and local disparities in coverage result from limited resources, competing health priorities, poor management of health systems, and inadequate monitoring and supervision.

Many health systems in SSA currently lack the capacity to provide even basic health care to the population, let alone deal with the additional burden of comorbidities from communicable and NCDs. This is particularly obvious when examining the availability of physicians, whose number per 100,000 inhabitants in 2004 was below the 1960 level in a number of countries (Tanzania, Zimbabwe, Liberia, Mozambique, and Sierra Leone). Elsewhere the number of physicians per 100,000 people stagnated over time or increased somewhat, but substantial increases between 1960 and 2004 were observed only in northern African countries and Mauritius.

The treatment gap in African countries has been mainly attributed to inadequately skilled personnel, cost of treatment, cultural beliefs, and unavailability of drugs, although lack of accessible health facilities has also been noted (50, 51, 91, 112, 114, 124–129). The lack of human resources and the difficulty retaining staff, especially in rural areas, is an important obstacle. A recent study found that alarming proportions of health care workers in African countries intend to migrate (129). The loss of health-care workers leaves crippling gaps in health-care systems of developing countries already struggling to combat mounting health crises, such as tuberculosis and AIDS (130, 131). Furthermore, while the bulk of social and health infrastructures and the best equipped hospitals are located in major cities in Africa, the African population is predominantly rural, with the urban population representing only 40 and 37%, respectively, of the total population in Africa and SSA in 2009.

## Assessing the relevance of the transition frameworks in Africa

### Testing the relevance of the demographic transition in Africa

To assess the extent of changes from 1950 to 2010 in mortality, fertility and population growth within the demographic transition perspective, we use two complementary approaches. First, graphical methods are used to assess trends in crude birth rates, crude death rates, infant and U5MR rates, total fertility rates, and life expectancy at birth from 1950 to 2010. We then evaluate whether the sequence of irreversible decline in fertility in response to mortality decline is occurring as expected from the demographic transition. Second, descriptive statistics are used to determine the ranges of variation (minimum, maximum) in estimates of crude birth rates, crude death rates, natural rates of population growth and total fertility rates. We then compare them to those expected when the demographic transition is/has taken place, as described above. These assessments are presented in Table 1.

**Table 1:** Assessing the demographic, epidemiological and health transitions in Africa in comparative perspective: 1950 to 2010

Major area, African region and country	CBR range of variation* 1950–2010	CDR range of variation* 1950–2010	RNI range of variation* 1950–2010	TFR Range of variation* 1950–2010	Onset of sustained fertility rate since 1950	e(0) 1950–1955	e(0) 2005–2010	e(0) average yearly change 1950–1990 (a)	e(0) average yearly change 1990–2010 (b)
World	20.1; 37.0	8.1; 19.1	12.0; 20.6	2.5; 5.0	1955	46.91	68.72	0.43	0.19
More developed regions	11.1; 22.4	9.4; 10.6	0.8; 11.8	1.6; 2.8	<1950	64.67	76.90	0.23	0.12
Less developed regions	22.0; 43.7	7.7; 23.1	14.4; 25.6	2.7; 6.1	1955	41.62	66.96	0.50	0.21
Sub-Saharan Africa	39.6; 47.4	13.0; 27.7	19.7; 28.9	5.4; 6.8	1980	36.23	52.94	0.32	0.15
Africa	36.7; 48.1	11.8; 26.8	21.3; 28.8	4.9; 6.7	1975	37.38	55.55	0.36	0.15
Eastern Africa	39.4; 49.3	11.1; 27.0	22.3; 30.3	5.4; 7.1	1980	37.03	55.85	0.30	0.27
Burundi	42.5; 51.4	14.2; 25.2	24.2; 33.8	6.5; 7.6	1980	39.03	51.32	0.24	0.10
Comoros	36.8; 47.4	9.6; 23.6	21.4; 31.9	5.1; 7.1	1985	40.72	59.66	0.36	0.19
Djibouti	28.9; 44.9	9.7; 21.0	19.2; 30.8	3.8; 6.8	1975	41.04	59.05	0.38	0.12
Eritrea	39.4; 48.0	8.0; 27.8	19.3; 31.2	5.2; 7.0	1960	35.84	59.95	0.27	0.54
Ethiopia	36.4; 49.3	9.5; 29.9	19.3; 30.3	5.3; 7.4	1955	34.08	59.26	0.30	0.53
Kenya	38.2; 51.5	10.0; 23.6	27.7; 37.7	4.8; 8.1	1970	42.30	57.23	0.43	−0.09
Madagascar	36.3; 49.1	7.9; 27.7	21.3; 31.5	4.8; 7.3	1975	36.32	62.23	0.34	0.49
Malawi	41.8; 55.0	13.5; 27.8	19.7; 33.5	5.8; 7.6	1985	36.32	51.56	0.26	0.19
Mauritius	12.4; 43.8	5.9; 15.4	5.3; 35.1	1.6; 6.2	1965	50.20	72.76	0.46	0.17
Mayotte	33.7; 49.4	2.4; 26.9	14.3; 42.3	4.3; 7.9	1980	47.26	77.94	0.60	0.27
Mozambique	42.3; 49.4	15.8; 32.8	16.5; 28.0	5.6; 6.6	1975	31.29	48.35	0.29	0.21
Réunion	18.2; 49.6	5.1; 19.2	13.1; 31.7	2.4; 6.9	1965	47.86	78.20	0.60	0.26
Rwanda	38.1; 53.8	9.1; 25.0	−0.6; 36.3	5.1; 8.4	1985	40.02	59.84	0.14	0.56
Seychelles	18.8; 39.0	7.1; 15.5	11.5; 27.4	2.2; 5.9	1970	57.96	72.41	0.33	0.05
Somalia	45.6; 49.7	13.7; 29.8	19.1; 33.2	7.1; 7.4	1970	33.99	53.21	0.31	0.27
South Sudan	38.5; 51.4	13.6; 37.3	10.4; 27.3	5.4; 6.9	1980	27.88	52.12	0.35	0.41
Uganda	45.8; 51.3	11.4; 24.5	26.8; 34.4	6.4; 7.1	2000	40.00	55.17	0.22	0.26
Tanzania	41.6; 49.3	10.8; 22.4	26.6; 31.5	5.6; 6.8	1975	41.25	56.60	0.25	0.22
Zambia	43.4; 49.3	13.7; 21.8	24.9; 32.6	5.9; 7.4	1980	42.07	50.94	0.10	0.20
Zimbabwe	31.9; 48.3	8.8; 17.4	14.4; 36.3	3.9; 7.4	1975	48.54	47.33	0.31	−0.54
Middle Africa	44.3; 47.7	15.8; 27.4	18.6; 29.7	6.0; 6.9	2005	36.77	49.53	0.27	0.08
Angola	48.2; 54.4	15.8; 35.8	18.2; 32.4	6.5; 7.4	1970	30.00	49.63	0.28	0.34
Cameroon	39.6; 46.4	13.2; 24.7	17.4; 31.3	5.2; 6.7	1985	38.54	52.73	0.37	−0.02
Central African Republic	35.8; 43.8	17.3; 31.0	10.7; 25.2	4.8; 6.0	1985	33.44	46.45	0.35	−0.03
Chad	46.1; 51.3	16.4; 28.6	17.8; 32.8	6.1; 7.4	2000	36.06	48.68	0.26	0.10
Congo	38.0; 43.1	11.8; 20.9	21.4; 29.8	5.1; 6.3	1980	43.16	55.68	0.33	−0.03
DR Congo	45.1; 48.8	16.9; 25.3	20.8; 30.7	6.0; 7.2	2000	39.06	48.27	0.20	0.04
Equatorial Guinea	32.9; 47.4	15.2; 30.4	10.5; 26.8	5.4; 5.9	2000	34.48	50.09	0.28	0.18
Gabon	30.3; 38.0	10.2; 27.7	2.6; 26.1	4.0; 5.7	1985	36.99	61.32	0.59	0.03
Sao Tome and Principe	35.8; 47.7	7.4; 21.0	27.0; 30.6	4.5; 6.5	1980	46.40	65.48	0.38	0.16
Northern Africa	24.8; 50.7	6.7; 23.5	18.0; 29.2	3.1; 6.9	1965	42.36	68.32	0.51	0.22
Algeria	19.3; 52.5	5.3; 23.7	14.0; 32.8	2.4; 7.6	1975	42.89	70.26	0.57	0.18
Egypt	24.3; 50.8	6.9; 25.3	17.4; 28.0	3.0; 6.7	1960	41.14	69.92	0.56	0.25
Libya	22.6; 50.8	4.2; 30.3	18.1; 34.8	2.7; 7.9	1980	36.67	74.18	0.77	0.27
Morocco	20.1; 51.5	6.1; 20.2	13.9; 32.2	2.4; 7.2	1965	45.67	69.66	0.44	0.26
Sudan	36.1; 46.9	8.9; 20.3	26.3; 32.7	4.8; 6.9	1980	44.54	60.90	0.27	0.23
Tunisia	16.9; 51.2	5.2; 24.5	11.6; 28.9	2.0; 7.1	1965	38.78	74.56	0.71	0.30
Western Sahara	22.6; 52.0	5.9; 27.9	16.7; 27.4	2.6; 6.6	1975	35.52	65.95	0.52	0.39
Southern Africa	22.8; 43.8	8.0; 20.4	8.0; 25.2	2.6; 6.3	1955	44.74	51.89	0.40	−0.36
Botswana	24.9; 47.3	7.1; 18.8	7.8; 35.1	2.9; 6.7	1970	47.66	46.53	0.40	−0.68
Lesotho	28.2; 42.7	9.9; 22.7	10.9; 26.8	3.4; 5.9	1975	42.16	45.59	0.38	−0.47
Namibia	28.1; 44.2	8.7; 22.9	18.3; 31.6	3.4; 6.6	1980	41.75	60.05	0.47	−0.02
South Africa	22.1; 43.7	7.9; 20.2	7.3; 24.7	2.6; 6.3	1955	45.01	52.21	0.40	−0.35
Swaziland	31.4; 49.3	9.5; 22.6	16.0; 35.7	3.8; 6.9	1975	41.42	47.35	0.43	−0.45
Western Africa	41.2; 47.8	13.6; 30.1	16.8; 28.4	5.7; 6.9	1985	33.75	52.32	0.35	0.18
Benin	39.1; 47.2	10.3; 34.5	6.3; 32.0	5.3; 7.0	1985	33.71	58.20	0.45	0.26
Burkina Faso	43.8; 49.0	12.8; 31.9	15.2; 31.0	6.1; 7.2	1985	30.94	53.97	0.46	0.18
Cape Verde	21.9; 48.9	5.3; 21.8	16.7; 32.2	2.6; 7.0	1970	48.07	73.16	0.43	0.32
Côte d'Ivoire	36.1; 54.3	13.6; 32.0	20.0; 33.3	4.9; 7.9	1975	32.14	48.71	0.52	−0.16
Gambia	43.6; 51.6	10.5; 32.7	10.9; 33.2	5.3; 6.3	1985	30.25	57.45	0.53	0.24
Ghana	33.1; 47.3	9.6; 21.1	23.6; 30.6	4.2; 7.0	1970	42.17	60.02	0.33	0.19
Guinea	39.2; 47.4	12.7; 33.2	13.9; 29.4	5.4; 6.6	1990	33.08	54.47	0.37	0.26
Guinea-Bissau	39.4; 54.5	13.5; 25.4	15.1; 32.9	5.3; 7.4	1955	40.58	52.99	0.19	0.19
Liberia	38.6; 49.2	10.3; 30.5	16.5; 29.8	5.2; 7.0	1985	33.12	58.11	0.36	0.43
Mali	47.7; 49.4	14.9; 38.3	10.2; 33.1	6.5; 7.1	1990	26.96	52.71	0.46	0.30
Mauritania	36.0; 49.8	9.2; 23.8	24.9; 30.3	5.0; 6.8	1975	38.59	60.66	0.48	0.12
Niger	50.4; 56.6	12.7; 28.1	26.6; 37.7	6.9; 7.8	2000	34.97	55.64	0.19	0.52
Nigeria	42.2; 47.1	14.9; 29.6	16.5; 27.6	6.0; 6.8	1985	34.01	50.18	0.30	0.16
Senegal	38.8; 50.5	8.3; 29.0	20.7; 32.6	5.1; 7.5	1980	35.47	62.23	0.51	0.25
Sierra Leone	39.8; 49.3	18.7; 34.9	9.2; 24.0	5.2; 7.0	1985	28.81	43.97	0.26	0.19
Togo	38.0; 48.3	11.8; 30.0	17.5; 32.3	4.9; 7.3	1980	35.30	54.73	0.50	−0.03

Source: Author's derivations from calculations based on online datasets from United Nations, Department of Economic and Social Affairs, Population Division (2013). World Population Prospects: The 2012 Revision, DVD Edition. New York: United Nations.

Notes: CBR = crude birth rate (births per 1,000 population); CDR = crude death rate (deaths per 1,000 population); RNI = rate of natural increase (per 1,000 population); TFR = total fertility rate (number of children per woman); e(0) = life expectancy at birth.

* Range of variation=(Minimum value of the indicator over the past 60 years; Maximum value of the indicator over the past 60 years).

(a) = Average yearly gain (+) or loss (−) in life expectancy at birth over the period 1950–1955 to 1985–1990;

(b) = Average yearly gain (+) or loss (−) in life expectancy at birth over the period 1985–1990 to 2005–2010.

#### Regional patterns of change in vital rates in Africa in comparative perspective between 1950 and 2010

**Fig. 1:**
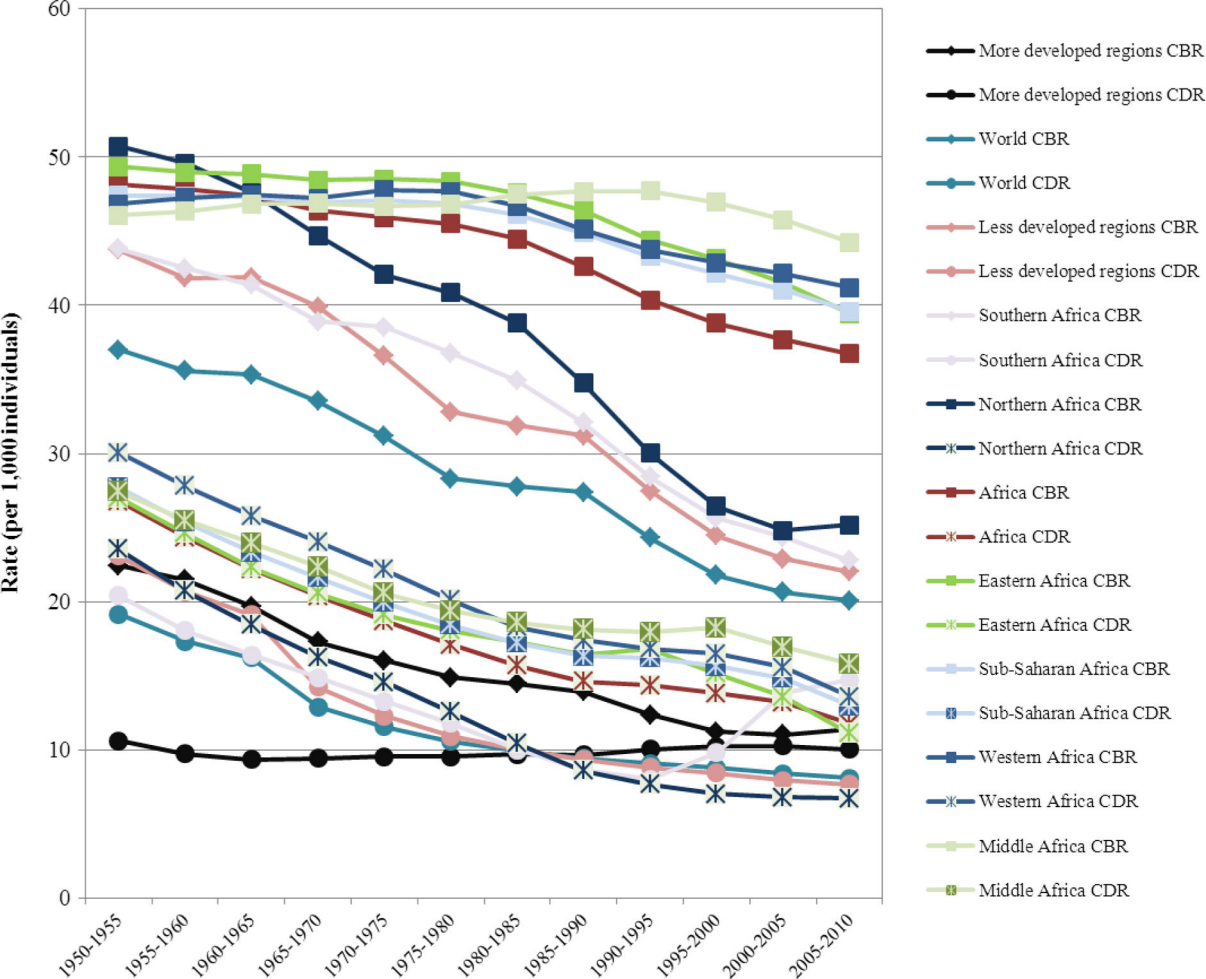
Crude birth and death rates for Africa and its regions, and other regions of the World, 1950–2010.

One approach to understanding the changes that have taken place in African population over the past 60 years is to consider the differences in major demographic indicators between then and now, using the latest and best available estimates from the United Nations’ online databases. Over the past six decades the population of Africa has witnessed a series of changes in population size and growth patterns due largely to variations in the two principal components of growth, that is, mortality and fertility. As during the demographic transition in Europe where fertility decline followed mortality decline in general, the rate of natural increase during the transitional period is expected to range between 0.5 and 1% per year in contemporary societies experiencing demographic transition (hypothesis 1). Trends in crude birth rates and crude death rates in Africa and across its regions between 1950–1955 and 2005–2010 are depicted in Fig. 1. Table 1 shows that the crude birth rates all remain very high and that with declining mortality though the levels are still very high, the range of variation in rates of natural increase has uniformly exceeded unity by a wide margin in all African regions and countries and over time (the one-time negative value for Rwanda is due to the mortality crisis during Rwandan genocide). Hence, we reject this hypothesis.

Between 1950–1955 and 2005–2010 crude birth rates and death rates both declined in Africa and its regions, although at a slower pace than in other regions of the world. Crude birth rates were higher in Northern Africa than SSA through the 1960s but have been higher in SSA than in Northern Africa ever since. Crude death rates, by contrast, have been consistently higher in SSA than in Northern Africa over time. Within SSA, differences across regions have varied over time; since the 1980s, crude birth rates and crude death rates have been highest in Middle Africa.

Over the past 50–60 years, major demographic changes have affected all regions and countries. The population growth rate in Africa rose from 2.11% in 1950–1955 (which was slower than that of Latin America, 2.72%, or of Oceania, 2.22%) to 2.30% in 2005–2010, which was the fastest rate in the world. Regional differences in population growth rate across Africa have been remarkable during the last 60 years. The growth rate declined from 2.34 to 1.74% in northern Africa, for example, but rose from 2.05 to 2.45% in SSA. Within SSA, southern Africa has experienced the sharpest decline in the rate of growth (from 2.30% in 1950–1955 to 1.02% in 2005–2010), while Middle Africa went from the lowest rate in 1950–1955 (1.93%) to the fastest rate in 2005–2010 (2.66%) in part due to a decline in infertility from controlling reproductive tract infections (132). Population growth has three major consequences for infectious disease: Growing populations in poor urban areas increase contact rates, facilitating epidemics; the sheer scale of cities provides more opportunities for a disease to persist with a ready supply of susceptible people; and the conditions associated with increases in travel and migration can turn epidemics into pandemics (133).

The world's population is shifting towards urban areas, with an estimated 51.6% living in urban areas in 2010 compared with 29.4% in 1950. By contrast, in 2010 only 39.2% of the population in SSA were urban dwellers. Due in large part to a diet linked with NCDs, urbanization is now linked to higher mortality rates for heart disease and chronic NCDs in poor countries (79–81, 134). Stuckler (135) found that each 1% increase in the population living in urban settings increased the long-term growth of chronic NCDs in the population by 3.2%.

Median age is a good summary of how young or old a population is. Half of the population in SSA is under 19 years, and half of the African population is under 20 years, indicating that these populations are extremely young. Population growth combined with the young populations typical of African countries and poorly resourced communities are influential factors of global health and human health in Africa (136).

The proportion of the population aged 60 years or older is lower in Africa than anywhere else in the world, ranging from 4.5% in middle Africa to 7.4% in northern Africa. Even if no changes were to occur in the age-specific risks of dying due to NCDs, population growth and aging in Africa would still produce large increases in the burden of mortality due to NCDs. The 2001 South African population census reported that 7.3% of the population was aged 60 years and older, and it is projected that even without changes in the risk factor profile or the mortality rates from cardiovascular disease, this demographic change will result in a doubling of the number of cardiovascular deaths in South Africa by 2040 (44).

It is conjectured that mortality decline will take place first, followed by fertility decline occurring 50 or more years from the onset of sustained fall in mortality. Levels of fertility and infant mortality are higher in Africa than other regions of the world, and within Africa the highest rates of fertility and infant mortality are in middle Africa. On average over the last 60 years, gains in life expectancy have also been lower in African regions (and lowest in middle Africa) than in the other regions of the world. During the transitional stage, the onset of mortality decline to moderate levels is expected to be followed after a lag time by fertility decline to less than five children per woman (hypothesis 2). It is unknown when mortality started declining in many African countries, since comparable data are available only since 1950.

#### Fertility rates in African countries and regions over the last 60 years in comparative perspective

Out of all African countries considered in this analysis using the latest and best available estimates from the United Nations’ online databases, a total fertility rate of five children or more per woman was seen in 23 of them, concentrated in three regions of Africa: 11 of the 16 countries of Western Africa (with Niger having the highest fertility rate, more than seven children per woman, in 2005–2010), eight of the 16 countries of Eastern Africa, and four of the nine countries of Middle Africa. For infant mortality, 11 countries (three in Western Africa, five in Middle Africa and three in Eastern Africa) had rates higher than 100 deaths per 1,000 live births in 2005–2010. Finally, 14 countries (three in Western Africa, two out of five in Southern Africa, five in Middle Africa, and four in Eastern Africa) had life expectancies at birth that were less than 50 years in 2005–2010.

**Fig. 2:**
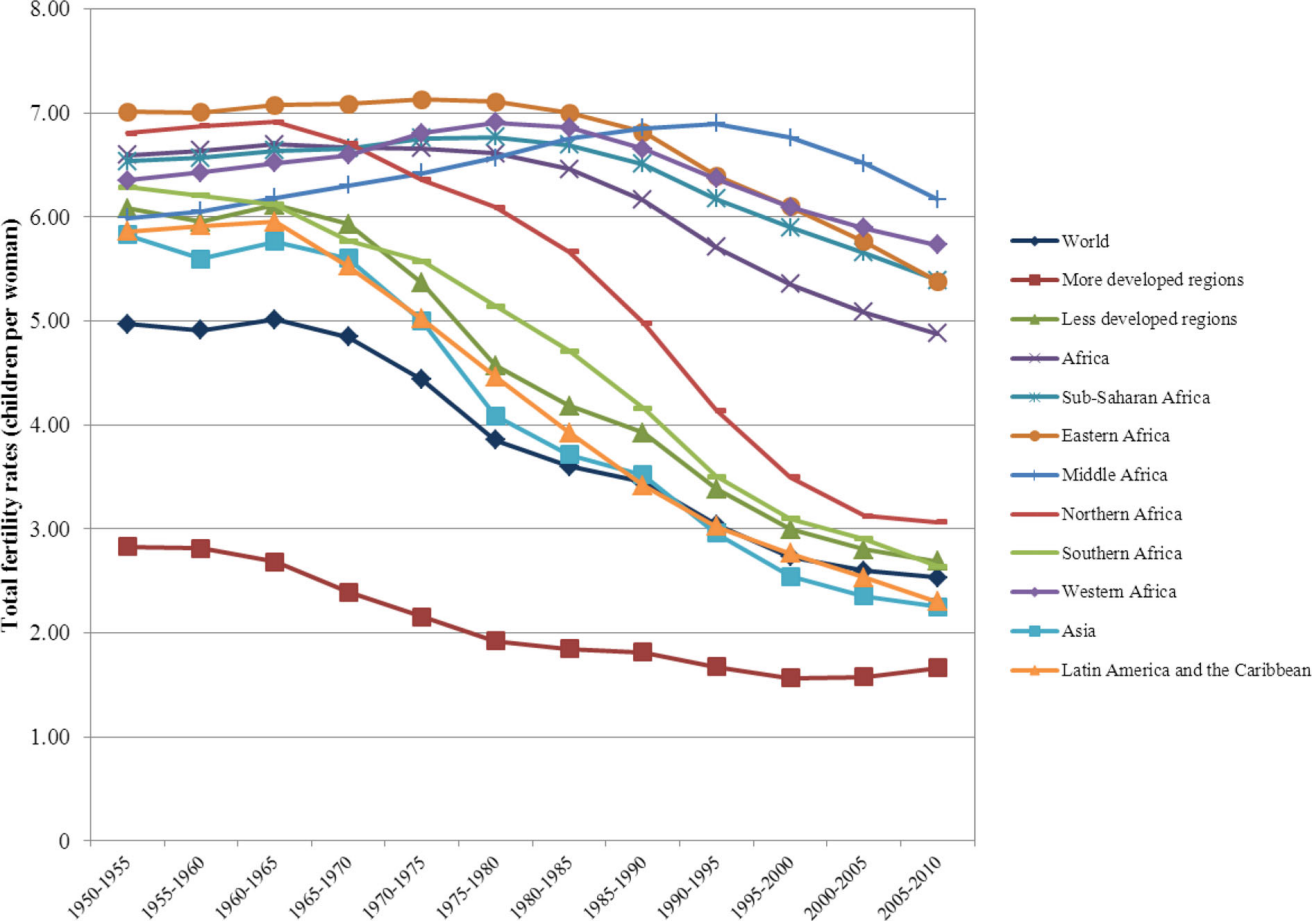
Total fertility rates for Africa and its regions, and other regions of the World, 1950–2010.

Figure 2 displays the patterns of fertility change in Africa and African regions in comparison to other regions of the world. Between 1950–1960 and 1990, fertility levels changed little and by 1990, Africa was the continent with the highest levels of fertility. Sub-Saharan countries seem to remain more systematically outside any established and uniform process of fertility change. For Africa and Sub-Saharan Africa, total fertility rates stand respectively at 4.9 and 5.4 children per woman in 2005–2010, compared to 6.7 and 6.8 children per woman in 1950–1955. In 2005–2010, the corresponding figures are 2.3 for Asia and 2.3 for Latin America and the Caribbean. The timing of the onset and pace of any decline of fertility decline have been quite variable over the past six decades in Africa, and signs of any decline occurred as late as the 1990s in many countries of middle Africa and western Africa. Furthermore, the pace of decline has been slowest in the regions of Sub-Saharan Africa than elsewhere. The many causes of these lowest rates in Sub-Saharan Africa reflect the familiar cultural, social, and economic constraints that impact women's reproductive health negatively in general. The region of the world where access and choice of contraception is still problematic and will remain so for a long time is sub-Saharan Africa.

Despite substantial reductions in mortality rates and increases in life expectancy over the past 60 years, many African countries and regions have maintained high fertility rates. There are important regional differences within the continent, with Middle Africa having the slowest progress in health improvements and maintaining high and often increasing fertility levels. Across Africa, the pace at which changes have occurred and the factors influencing them has varied from one group of countries to another. With the exception of Mauritius, which shows a consistent pattern of changes over time, there is little evidence of a pattern in which the timing of changes in mortality can be linked to the timing of changes in fertility, and, indeed, most countries have achieved substantial reductions in mortality and gains in life expectancy at birth with virtually no variations in fertility. Despite what would be expected according to the frameworks of the demographic transition, the epidemiological transition, and the health transition, mortality declines in African nations were generally not followed by fertility declines, even after 60 years of scrutiny (2, 41, 137); these results substantiate arguments for what several scholars have termed ‘African exceptionalism’ (68–70), given Africa's departure from the prediction of the transition from high to low fertility in response to mortality decline inherent in demographic and epidemiological transitions. Northern and Southern Africa and the island countries (Mauritius, Reunion, Seychelles, Djibouti, and Cape Verde) are exceptions, but their transitions generally predated the 1960s. Although fertility decline started in SSA in 1980 and in Africa since 1975, fertility rates are still above the expected transition-level of five children per woman in many African regions (notably Middle, Eastern, and Western Africa). Hence, hypothesis 2 is not confirmed.

In short, the patterns of changes seen in Africa do not in general adhere to the predictions of the various transition frameworks, and the only places in SSA where these frameworks do apply are island countries such as Mauritius. The vast majority of the other African countries moved from a homogeneous pattern of populations with uniformly high levels of mortality and fertility prior to the independence years in the 1960s to a heterogeneous pattern of populations with widely varying rates of fertility, infant mortality, and life expectancy. This is in direct contrast to the expectations of the various transition frameworks, which conjecture populations that become more homogeneous over time with respect to patterns of mortality and fertility as well as life expectancy trajectories. We conclude that, with respect to mortality and fertility patterns, these frameworks do not perform well in accounting for the timing and sequencing of mortality decline and fertility decline since these are highly variable in Africa across African countries and regions.

We also compute the average yearly gains/losses in life expectancy between the 1950–1955 and 1985–1990 and between 1985–1990 and 2005–2010, to explore the impact of HIV/AIDS epidemic on trends in life expectancy in African countries. We then compare these changes to those expected by the demographic transition. During the transitional stage (like from 1800 to 1950 in Europe), life expectancy at birth is expected to array between 40 and 65 years (hypothesis 3). Table 1 shows improvements in life expectancy for all countries through the 1980s. Since the 1990s, there are signs of stagnation or fall in life expectancy below the pre-1990 level. Such fall has been palpable in all countries from Southern Africa, in some countries of Middle Africa (Cameroon, Central African Republic, and Congo) or Eastern Africa (Kenya and Zimbabwe) and Western Africa (Côte d'Ivoire and Togo). Moreover, several countries have witnessed declines in life expectancy at birth between 1990 and 2010, falls which bring their life expectancy close to the 1950–1955 levels where some countries had life expectancy at birth under 40 years in 1950. Therefore, the pattern of change in life expectancy in Africa has been greatly influenced by the HIV/AIDS epidemic and among other influential factors, and hypothesis 3 is not accepted.

Overall, the evidence fails to confirm the conjectures of the demographic transition in Africa. These assessments also apply to shared features among the demographic, epidemiological and health transitions.

### Testing the relevance of the epidemiological transition in Africa

We focus on the first three propositions which describe the epidemiological transition. We use reviewed evidence for the fourth and fifth propositions which are concerned with its determinants. The first proposition relating to the relative role of mortality and fertility in the epidemiological transition has been tested above in the context of the assessment of the demographic transition.

#### Distribution and group ratio of cause-specific deaths in Africa in comparative perspective

The second proposition is that during the transition, there is a long-term shift in cause-of-death patterns whereby NCDs predominate as primary cause of death. We compare the causes-of-death pattern in Africa with other parts of the world, using: 1) the ratio of group II causes-of-death (NCDs) to Group I causes-of-death (communicable, maternal, perinatal and nutritional conditions); and 2) the distribution of the top 20 causes of death to appraise the weight of mortality from communicable diseases and the expected changes in life expectancy. The ratio of Group II to Group I causes of deaths gives an indication of the process of the epidemiological transition: the higher the ratio, the more the epidemiological transition is advanced in a society. Tables 2 and 3 show the distribution of causes of death and ratios of causes of death.

**Table 2:** Percent distribution and group ratio of cause of deaths in the world, in the developed and developing regions, and in Africa: 2008

	World	Developed regions	Africa	Latin America and the Caribbean	Eastern Asia	Southern Asia
All causes	100	100	100	100	100	100
Group I. Communicable, maternal, perinatal, and nutritional conditions	27	7	65	15	7	38
Group II. Non-communicable diseases	64	87	28	73	83	52
Group III. Injuries	9	6	7	12	10	10
Ratio of Group II to Group I deaths	2.4	12.4	0.4	4.9	11.9	1.4

Source: Author's calculations based on online World Health Organization’s regional cause-specific mortality estimates for the year 2008. (http://www.who.int/healthinfo/global_burden_disease/en/).

**Table 3:** Percent distribution of the top 20 cause of deaths in the world, in the developed world, in developing regions, and in Africa: 2008

Top 20 causes of death: world, 2008		Top 20 causes of death: developed world, 2008		Top 20 causes of death: Africa, 2008	

1. Ischemic heart disease	12.75	1. Ischemic heart disease	15.86	1. HIV/AIDS	12.87
2. Cerebrovascular disease	10.81	2. Other cardiovascular diseases	9.01	2. Lower respiratory infections	11.18
3. Lower respiratory infections	6.09	3. Cerebrovascular disease	8.58	3. Diarrheal diseases	9.08
4. Chronic obstructive pulmonary disease	5.76	4. Trachea, bronchus, lung cancers	5.97	4. Malaria	7.47
5. Diarrheal diseases	4.33	5. Alzheimer and other dementias	4.22	5. Cerebrovascular disease	4.43
6. Other cardiovascular diseases	3.77	6. Lower respiratory infections	3.83	6. Tuberculosis	3.93
7. HIV/AIDS	3.12	7. Chronic obstructive pulmonary disease	3.58	7. Ischemic heart disease	3.70
8. Trachea, bronchus, lung cancers	2.44	8. Other malignant neoplasms	3.39	8. Prematurity and low birth weight	3.31
9. Tuberculosis	2.36	9. Colon and rectum cancers	3.31	9. Birth asphyxia and birth trauma	2.93
10. Road traffic accidents	2.12	10. Other digestive diseases	2.65	10. Other cardiovascular diseases	2.74
11. Hypertensive heart disease	2.03	11. Hypertensive heart disease	2.14	11. Neonatal infections and other conditions	2.52
12. Other unintentional injuries	2.01	12. Other respiratory diseases	2.13	12. Road traffic accidents	1.66
13. Other malignant neoplasms	1.88	13. Breast cancer	1.97	13. Other digestive diseases	1.64
14. Other digestive diseases	1.82	14. Nephritis and nephrosis	1.67	14. Meningitis	1.62
15. Prematurity and low birth weight	1.75	15. Pancreas cancer	1.63	15. Violence	1.60
16. Cirrhosis of the liver	1.49	16. Stomach cancer	1.57	16. Childhood-cluster diseases	1.38
17. Neonatal infections and other conditions	1.46	17. Self-inflicted injuries	1.52	17. Other unintentional injuries	1.35
18. Malaria	1.45	18. Prostate cancer	1.51	18. Other respiratory diseases	1.33
19. Self-inflicted injuries	1.37	19. Lymphomas, multiple myeloma	1.41	19. Chronic obstructive pulmonary disease	1.30
20. Birth asphyxia and birth trauma	1.37	20. Cirrhosis of the liver	1.41	20. Protein-energy malnutrition	1.05
All the other causes	29.82	All the other causes	22.64	All the other causes	22.94

Top 20 causes of death: Latin America, 2008		Top 20 causes of death: Eastern Asia, 2008		Top 20 causes of death: Southern Asia, 2008	

1. Ischemic heart disease	11.47	1. Cerebrovascular disease	21.61	1. Ischemic heart disease	12.88
2. Cerebrovascular disease	7.96	2. Chronic obstructive pulmonary disease	12.88	2. Diarrheal diseases	9.54
3. Lower respiratory infections	5.16	3. Ischemic heart disease	10.79	3. Chronic obstructive pulmonary disease	8.21
4. Other cardiovascular diseases	5.07	4. Trachea, bronchus, lung cancers	4.81	4. Cerebrovascular disease	7.92
5. Violence	4.30	5. Liver cancer	3.93	5. Lower respiratory infections	7.82
6. Hypertensive heart disease	3.51	6. Stomach cancer	3.67	6. Tuberculosis	3.30
7. Other digestive diseases	3.44	7. Road traffic accidents	3.00	7. Neonatal infections and other conditions	2.91
8. Other malignant neoplasms	3.22	8. Other cardiovascular diseases	2.34	8. Prematurity and low birth weight	2.70
9. Chronic obstructive pulmonary disease	3.11	9. Hypertensive heart disease	2.24	9. Birth asphyxia and birth trauma	2.20
10. Road traffic accidents	3.06	10. Other unintentional injuries	2.13	10. Road traffic accidents	2.03
11. Other respiratory diseases	2.56	11. Esophagus cancer	2.13	11. Other unintentional injuries	2.01
12. Cirrhosis of the liver	2.46	12. Lower respiratory infections	2.03	12. Self-inflicted injuries	1.89
13. Nephritis and nephrosis	2.04	13. Self-inflicted injuries	1.81	13. Childhood-cluster diseases	1.87
14. Other unintentional injuries	1.99	14. Other malignant neoplasms	1.76	14. Cirrhosis of the liver	1.85
15. Trachea, bronchus, lung cancers	1.83	15. Tuberculosis	1.69	15. Hypertensive heart disease	1.67
16. Stomach cancer	1.67	16. Colon and rectum cancers	1.24	16. Nephritis and nephrosis	1.61
17. HIV/AIDS	1.42	17. Cirrhosis of the liver	1.22	17. Other malignant neoplasms	1.57
18. Prematurity and low birth weight	1.34	18. Falls	1.20	18. Other digestive diseases	1.48
19. Prostate cancer	1.25	19. Other digestive diseases	1.12	19. HIV/AIDS	1.47
20. Colon and rectum cancers	1.16	20. Other respiratory diseases	1.09	20. Falls	1.46
All the other causes	31.98	All the other causes	17.31	All the other causes	23.61

Source: Author's calculations based on online World Health Organization’s regional cause-specific mortality estimates for the year 2008. (http://www.who.int/healthinfo/global_burden_disease/en/).

According to the Omran's epidemiological transition (11), mortality by cause of death in non-western countries was projected to shift in predominance from communicable diseases dropping from being responsible for 42.1% of all deaths in the less developed regions in 1970 to 19.4% in 2015, resulting in life expectancy at birth rising from 57.5 to 68.5 years (hypothesis 4). Mortality and cause of death patterns indicates that across the world, 27% of deaths are ascribed to communicable, maternal, perinatal and nutritional conditions. In developed regions that number is less than 7%. Africa stands far apart from the rest of the world, with 65% of deaths caused by these conditions; infectious and parasitic diseases alone are responsible for 41% of all deaths in Africa, compared with only 15% for the world as a whole. In sharp contrast, 64% of all deaths worldwide are due to NCDs; that figure is 87% in the developed regions and 28% in Africa. In most developing regions, noncommunicable diseases (Group II causes of death) already exceeded Group I causes of death in 1990, whereas the ratio was only 0.4 in SSA (67). Table 2 indicates that almost 20 years later, that ratio is still at 0.4 for Africa, compared with 2.4 for the world, 12.4 in developed countries, 11.9 in Eastern Asia, 4.9 in Latin America and the Caribbean, and 1.4 in Southern Asia. Hence, hypothesis 4 is not verified in Africa.

Table 3 presents the distribution of the 20 leading causes of death by region of the world. The list of the 20 leading causes of death in Africa highlights the double burden of increasing communicable and NCDs in Africa and contradicts the expectations of the epidemiological transition. Contrary to the expected shift between communicable and NCDs, with the former decreasing and the latter increasing, the emerging pattern of disease in the African context is one of increasing co-occurring morbidities from communicable and NCDs. The prevalence of old, emerging, or re-emerging communicable diseases such as tuberculosis, malaria, and HIV have been stable or increasing, while the levels of NCDs such as diabetes have been increasing (50, 51, 64, 91, 128, 138, 139).

**Fig. 3:**
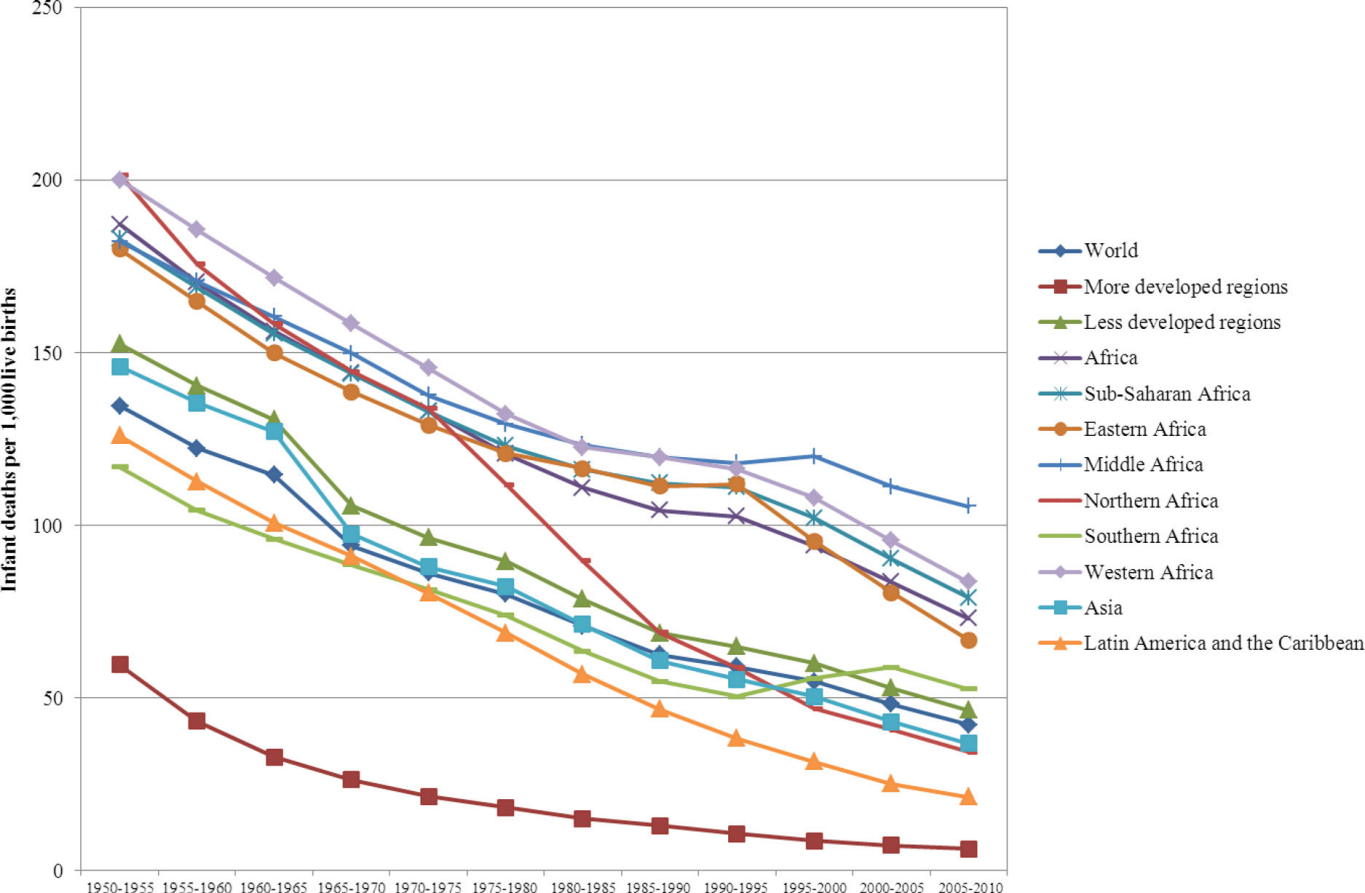
Infant Mortality Rates for Africa and its regions, and other regions of the World, 1950–2010.

**Fig. 4:**
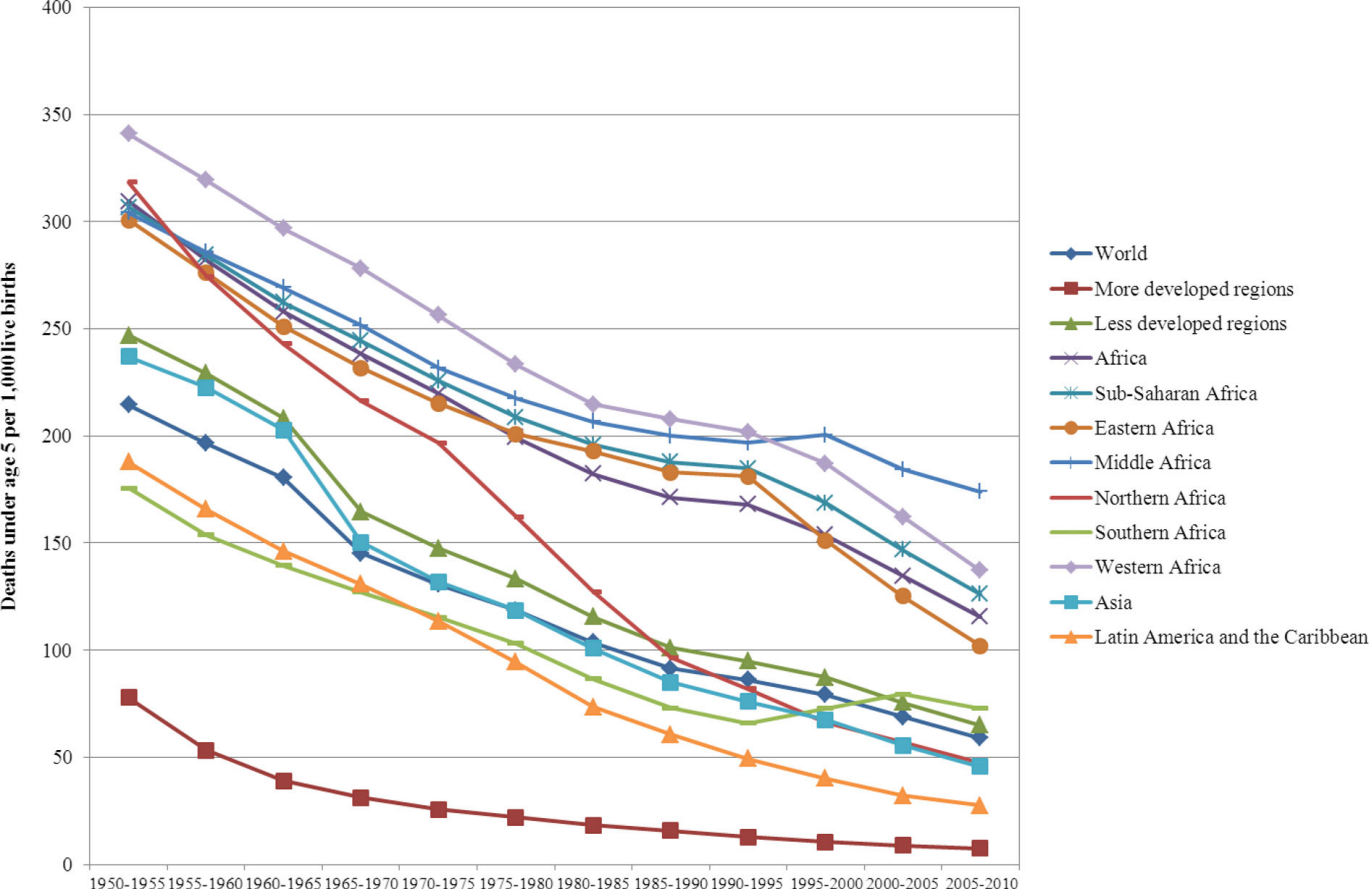
Under Five Mortality Rates for Africa and its regions, and other regions of the World, 1950–2010.

**Fig. 5:**
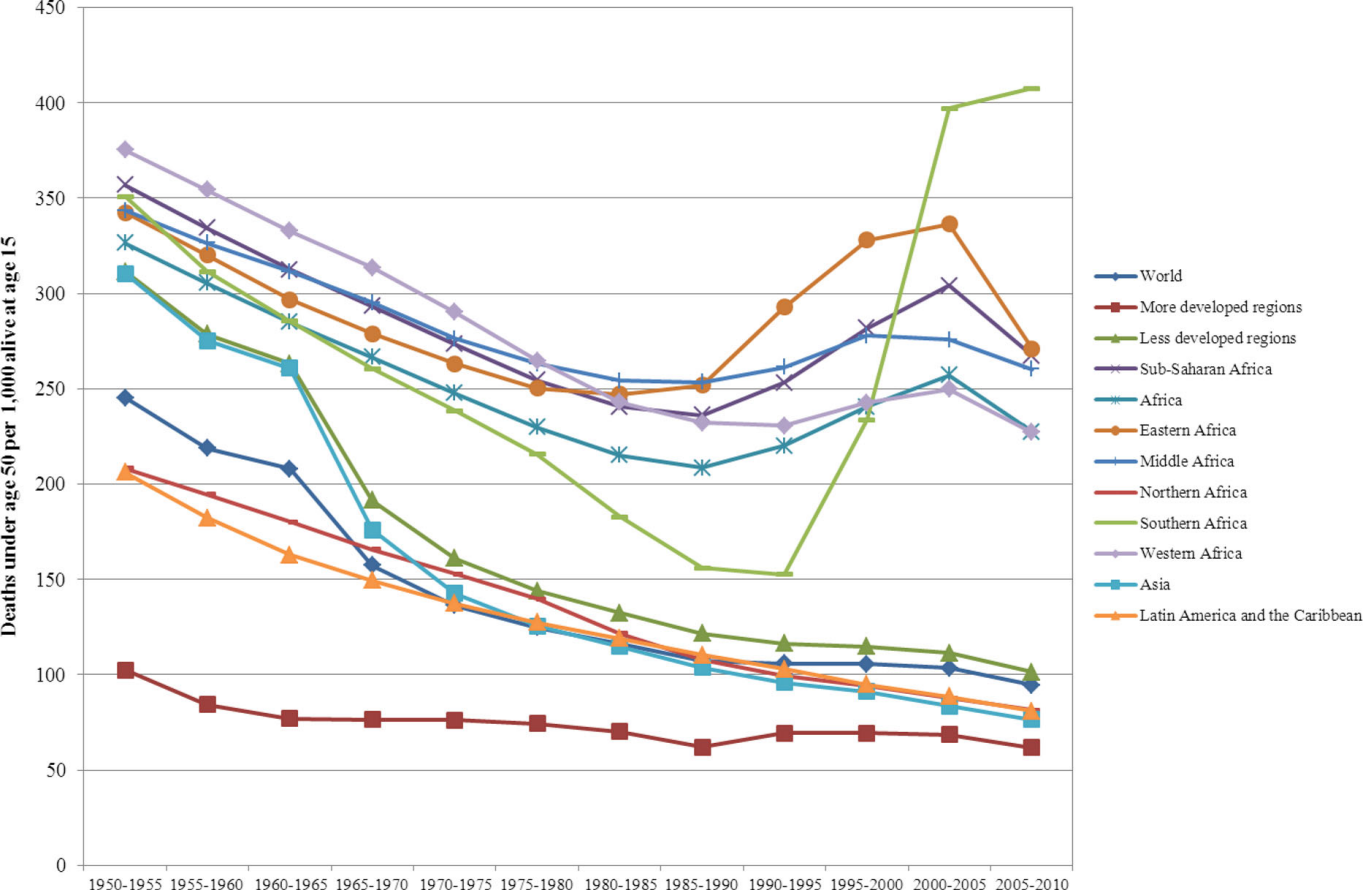
Adult Mortality for Africa and its regions, and other regions of the World, 1950–2010.

The third proposition is that there are age and sex differentials resulting from the epidemiological transition, with the transition usually favoring young over old people (hypothesis 5) and mortality decline over time is expected to be more advantageous to females than males (hypothesis 6). To test this proposition, we generate age-sex-specific estimates by calculating male-to-female mortality ratios over the 1950–2010 periods, for infant, child and adult mortality. We then evaluate changes over time in these ratios. To test hypothesis 5, falling trends in age-specific mortality rates are graphed on the same scale in Fig. 3 (for infant mortality), Fig. 4 (for U5MR) and Fig. 5 (for adult mortality). To test hypothesis 6, ratios of male to female of infant, child and adult mortality are presented in Table 4.

**Table 4:** Ratio of male to female (M/F) (per 100) of infant mortality rate (IMR), child mortality rate (CMR), and adult mortality rate (AMR) by major areas, African regions and countries: 1970–2010

	Ratio of M/F in the 1970s			Ratio of M/F in the 1980s			Ratio of M/F in the 1990s			Ratio of M/F in the 2000s		
	
Regions and African countries	IMR	CMR	AMR (a)	IMR	CMR	AMR (b)	IMR	CMR	AMR (c)	IMR	CMR	AMR (d)
World	111	90	128	110	89	138	108	87	144	107	86	142
More developed regions	129	123	230	129	122	232	128	124	255	126	124	244
Less developed regions	111	89	114	110	88	127	107	87	132	107	86	132
Sub-Saharan Africa	116	103	115	117	104	115	117	103	106	118	103	100
Eastern Africa			115			116			103			98
Burundi	115	99	115	115	99	117	115	99	106	115	98	107
Comoros	–	–	117	–	–	119	–	–	116	–	–	112
Djibouti	–	–	117	–	–	119	–	–	122	–	–	115
Eritrea	123	110	112	123	109	114	123	107	121	123	104	122
Ethiopia	122	109	114	124	107	113	125	103	103	126	97	100
Kenya	112	110	124	116	109	125	120	108	108	124	107	101
Madagascar	107	99	110	116	103	113	126	107	111	136	109	116
Malawi	112	104	111	112	107	115	111	110	102	110	115	86
Mauritius	126	98	207	131	114	212	133	116	234	131	115	232
Mayotte	–	–	186	–	–	210	–	–	235	–	–	263
Mozambique	117	98	114	114	102	114	111	106	113	108	110	102
Réunion	–	–	186	–	–	210	–	–	235	–	–	263
Rwanda	114	112	115	113	110	117	112	108	104	111	105	110
Seychelles	–	–	207	–	–	237	–	–	264	–	–	259
Somalia	116	108	114	116	108	115	116	108	117	116	107	117
South Sudan	–	–	112	–	–	113	–	–	111	–	–	104
Uganda	106	114	115	112	115	118	119	116	96	126	117	92
Tanzania	112	109	117	112	106	117	111	103	102	111	99	97
Zambia	112	91	119	114	100	109	116	108	95	118	117	97
Zimbabwe	134	94	122	124	102	127	115	114	101	105	129	90
Middle Africa			114			115			111			110
Angola	–	–	113	–	–	116	–	–	112	–	–	110
Cameroon	120	99	116	122	98	117	124	96	108	126	95	101
Central African Republic	113	99	119	114	101	123	114	103	109	115	105	104
Chad	115	108	110	116	105	112	118	102	106	119	99	103
Congo	119	95	118	119	94	119	119	95	105	119	95	105
DR Congo	106	112	114	106	112	114	106	112	114	106	112	116
Equatorial Guinea	–	–	114	–	–	116	–	–	112	–	–	110
Gabon	137	109	118	137	107	122	137	103	114	137	103	102
Sao Tome and Principe	–	–	119	–	–	126	–	–	128	–	–	133
Northern Africa			125			132			137			139
Algeria	104	82	112	109	96	121	114	107	123	119	132	123
Egypt	103	81	157	105	81	159	109	84	160	122	116	161
Libya	114	82	126	114	78	129	114	69	128	114	60	129
Morocco	112	88	113	116	95	120	120	96	124	125	98	126
Sudan	120	101	117	118	101	118	115	102	132	113	102	135
Tunisia	109	78	106	119	94	124	128	112	147	137	141	154
Western Sahara	–	–	114	–	–	115	–	–	117	–	–	125
Southern Africa			133			151			115			94
Botswana	132	111	121	132	110	133	132	110	114	132	113	95
Lesotho	112	129	110	112	129	111	112	128	103	112	128	96
Namibia	110	110	114	118	111	129	126	113	115	134	117	128
South Africa	126	105	135	124	112	154	122	120	117	119	129	94
Swaziland	127	95	116	127	94	115	127	93	105	127	97	90
Western Africa			111			110			106			103
Benin	115	102	128	114	98	129	113	94	118	111	88	112
Burkina Faso	120	111	116	115	104	114	111	98	120	106	92	103
Cape Verde	–	–	106	–	–	132	–	–	189	–	–	207
Côte d'Ivoire	123	110	118	125	108	122	126	108	101	127	107	105
Gambia	–	–	112	–	–	113	–	–	114	–	–	114
Ghana	117	96	113	120	102	111	123	111	107	126	119	106
Guinea	111	96	111	113	98	111	116	99	97	118	100	108
Guinea-Bissau	–	–	117	–	–	122	–	–	113	–	–	109
Liberia	123	111	116	120	109	123	118	107	97	115	104	107
Mali	117	99	110	116	101	93	115	105	89	114	108	86
Mauritania	112	91	116	116	100	112	121	105	115	126	111	115
Niger	114	92	104	113	94	99	112	97	94	111	99	95
Nigeria	116	103	110	116	101	110	116	100	105	116	98	102
Senegal	117	97	112	117	101	120	118	106	118	119	113	117
Sierra Leone	–	–	91	116	115	96	114	115	106	112	115	98
Togo	125	107	118	125	104	118	125	102	103	125	98	103

Source: Author's derivations from calculations based on online datasets from United Nations, Department of Economic and Social Affairs, Population Division (2013). World Population Prospects: The 2012 Revision, DVD Edition. New York: United Nations.

Notes: IMR = infant mortality rate; CMR = child mortality rate; AMR = adult mortality rates (probability of dying between exact ages 15 and 50). (a) = 1975–1980; (b) = 1985–1990; (c) = 1995–2000; (d) = 2005–2010.

#### Infant mortality in African countries and regions over the last 60 years in comparative perspective

Figure 3 shows the changing trends of infant mortality rates across regions in Africa since 1950, in comparison to other regions of Africa and the World. Infant mortality rates, often used as a key indicator of the well-being of a population, have been uniformly higher in Africa than any other continent of the world since World War II. Within the African continent, three patterns emerge. First, of the five regions of Africa, infant mortality was highest in Western Africa from 1950 to 1955 through 1985–1990; since then, middle Africa has had the highest infant mortality rates in the continent. Second, infant mortality rates were higher in northern Africa than SSA through the 1960s. Third, infant mortality rates were consistently lowest in southern Africa until the mid-1990s, when northern Africa became the region of the continent with the lowest infant mortality rates; AIDS-related deaths triggered by mother-to-child transmission of HIV caused infant mortality rates in southern Africa to rise between 1995–2000 and 2000–2005. There have also been huge differences in levels and trends in infant mortality rates across countries within Africa. South Africa had the lowest infant mortality rates in 1950–1955 (96 deaths per 1,000 live births) and Burkina Faso the highest (308 deaths per 1,000 live births). By 2005–2010 Mayotte and Reunion had the lowest rates, at six deaths per 1,000 live births, while Chad had the highest, with 131 deaths per 1,000 live births. Many African countries have maintained unacceptably high levels of infant mortality for over half a century and levels have stalled in a number of countries since the 1990s.

A major premise of the epidemiological transition is that mortality is the fundamental factor in population dynamics. However, between 1950–1960 and 1990, fertility levels in Africa changed little, and by 1990 Africa was the continent with the highest levels of fertility. In 2005–2010 total fertility rates in Africa were 4.88 children per woman, compared to 6.6 children per woman in 1950–1955; the corresponding estimates for SSA were 5.39 for 2005–2010 and 6.53 for 1950–1955, therefore situating sub-Saharan African fertility pattern using the latest and best estimates from the United Nations still at pre-transition levels (see Table 1). In 2005–2010, the corresponding figures were 2.3 for Asia and 2.3 for Latin America and the Caribbean. The timing of the onset and the pace of any fertility decline have been quite variable across Africa over the past 60 years, and in many countries of middle Africa and western Africa signs of a fertility decline did not start appearing until the 1990s. The pace of fertility decline has been slower in SSA than anywhere else in the world because of cultural, social, and economic constraints that impact women's reproductive health negatively in general (66, 138).

#### U5MR and adult mortality in African countries and regions in comparative perspective

Figures 4 and 5 show the changes in levels and pace of variations of U5MR and adult mortality in Africa and its individual regions in comparative perspective with other regions of the world. The situation in Africa is quite different from other regions of the world, where there is unquestionable evidence of regular mortality reductions over time in all regions (with the notable exception of eastern Europe, where adult mortality rate rose from 247 in 1995–2000 to 258 in 2000–2005, a divergent trend from the rest of Europe and developed world). In particular, as is the case with the fertility patterns discussed above, under-five and adult mortality patterns in sub-Saharan African countries do not adhere to the standard demographic, epidemiological, or health transitions. There are a variety of reasons for this. One is the widespread presence of wars and other forms of social and political unrest in the continent. A second is the emergence or re-emergence of such infections as HIV/AIDS and tuberculosis.

Perhaps the best way to observe the effect of wars and other conflicts on mortality rates across Africa is to examine those rates on a more local level. Indeed, local statistics in conflict areas indicate that mortality reductions would certainly have been deeper in the absence of wars in Africa.

The impact of wars is especially apparent in the data on U5MR in eastern African and middle African countries. In eastern Africa in the 1990–1995 period, five countries experienced reversals in mortality trends due to rampant wars: Rwanda, Burundi, Somalia, Zambia, and Uganda. In middle Africa wars have spanned several periods, and U5MR has increased for more than 10 years in several countries, notably Chad, Central African Republic, Congo, and Democratic Republic of Congo. In western Africa, wars reversed trends in U5MR rate in Sierra Leone, Liberia, and Côte d'Ivoire.

Similarly, AIDS has impacted and reversed trends in both U5MR and adult mortality in several countries in southern Africa, eastern Africa, middle Africa, and western Africa; in contrast, trends in mortality decline among children and adults have been robust in northern Africa (as well as the islands of Mauritius, Mayotte and Reunion, which by all accounts are apart in terms of population change and epidemiological landscapes from the rest of Africa). Since the period 1995–2000, every country in southern Africa (with the exception of Namibia which has singled itself out as the sole southern African country with a sustained decline in U5MR over time) has experienced increases in both U5MR and adult mortality due to the HIV/AIDS epidemic. These increases have been huge and sustained for adult mortality rates, with increases ranging from 25.9% in Namibia to 46.8% for Lesotho. South Africa has been particularly affected, and it is the only southern African country that has experienced continued increases in adult mortality rates since the 1990s, jumping from 369 in 1995–2000 to 490 in 2000–2005 and 533 in 2005–2010. U5MR rates for the whole of southern Africa increased substantially from 66 per 1,000 live births in 1990–1995 to 73 in 1995–2000 and 79 in 2000–2005.

In Eastern Africa, AIDS led to a reversal in the trends of decreasing mortality rates especially for adults. In middle Africa, adult mortality rates increased between 1995–2000 and 2000–2005 in all countries but Congo and Democratic Republic of Congo. The effects of AIDS on mortality rates are most obvious in Cameroon and Gabon, neither of which has been engaged in wars for decades: In Cameroon, for example, U5MR rate rose from 150 in 1985–1990 and 1990–1995 to 155 in 1995–2000 and 157 in 2000–2005 and remained at 152 in 2005–2010, and adult mortality rate increased from 370 in 1995–2000 to 412 in 2000–2005 and 413 in 2005–2010. In Gabon adult mortality rates increased from 270 in 1995–2000 to 307 in 2000–2005 and 286 in 2005–2010.

On the other hand, since 2005 Africa has been experiencing some of the largest decreases in U5MR ever seen anywhere. Many individual African countries have experienced declines in their U5MR rates, with most of these decreases having been 4.4% or more yearly, which is the rate of decline needed to meet the Millennium Development Goal of cutting by two-thirds the child-mortality rate between 1990 and 2015. The striking thing about the decreasing rates is how widespread they have been, occurring in all countries and regions of the continent.

These mortality situations and patterns are sharply different from the historical situations experienced in developed countries and are also in disconnect with the patterns predicted by the standard demographic, epidemiological, and health transition frameworks. Therefore, hypothesis 5 is rejected.

In Table 4, we test the hypothesis that during the epidemiological transition, mortality decline over time is more advantageous to females than males (hypothesis 6). The mortality rates considered refer to the infant mortality rates (the probability of dying between birth and exact age 1 year), the child mortality (the probability of dying between exact ages 1 and 5 years), and the adult mortality (the probability of dying between exact ages 15 and 50 years). Despite falls in mortality, sex mortality differentials and male disadvantage has been noted whereby boys tend to have higher mortality rates than girls at early ages, leading to male-to-female mortality ratios expected to be greater than 100. The sex ratio of child mortality is generally lower than the sex ratio of infant mortality. During the epidemiological transition, it is expected that improvements in living conditions will make infectious diseases recede as a cause of death. On the one hand, as this occurs, perinatal and congenital causes form an increasing share of total mortality among infants; since newborn girls tend to be less vulnerable to such perinatal conditions (including birth trauma, intrauterine hypoxia and birth asphyxia, prematurity, respiratory distress syndrome and neonatal tetanus) and congenital anomalies, this will affect more boys than girls and male-to-female mortality ratio will rise because of this male disadvantage. On the other hand, external causes more typically affecting boys, form an increasing share of mortality for children between ages 1 and 5. Hence, as overall levels of mortality fall, female advantage in infant and child mortality would normally increase assuming no sex-specific changes in the treatment of children.

In Table 4, while there is little change over time in sex ratio of infant mortality or child mortality in most countries, excess female child mortality can be found notably concentrated at various periods of time in Northern Africa (Algeria, Egypt, Libya, Morocco and Tunisia), Western Africa (Benin, Burkina Faso, Ghana, Guinea, Mali, Niger and Senegal) and Middle Africa (Cameroon, Congo). Previous studies have signaled sex differential treatment in some of these countries but not in other (138). There is also a relative stability of male to female adult mortality ratios over time across countries, with notable exceptions of high-HIV countries of Eastern Africa (Malawi, Uganda, Tanzania, Zambia and Zimbabwe) and Southern Africa (Botswana, Lesotho, South Africa and Swaziland) where there is a consistent pattern of excess female mortality in recent periods most likely due to AIDS mortality. These results provide no evidence consistent with the conjectures from the epidemiological transition; hence, we reject hypothesis 6.

### Testing the relevance of the health transition in Africa

All of the assessments above apply here. In the health transition view, formal education in general and female education notably is often cited as the most powerful instrument that has been shown to accelerate social changes enhancing health and improving life expectancies. Concerning sex differentials, mortality decline over time is expected to be more advantageous to females than males. In Western countries, female age-specific mortality risks shifted from being higher than those for the male in age groups under 45 to lower risks than the male in all age groups as life expectancy reached 60 or 70 years. Since improvement in survival is expected to favor females during the course of the epidemiological transition, sex differences favoring female populations in life expectancies at birth, at age 15 and at age 60 suggest the occurrence of health transition (hypothesis 7). These age-specific sex differentials in life expectancy are used to determine if there is any pattern of change consistent with these predictions from the health transition perspective.

#### Life expectancy in African countries and regions over the last 60 years in comparative perspective

**Fig. 6:**
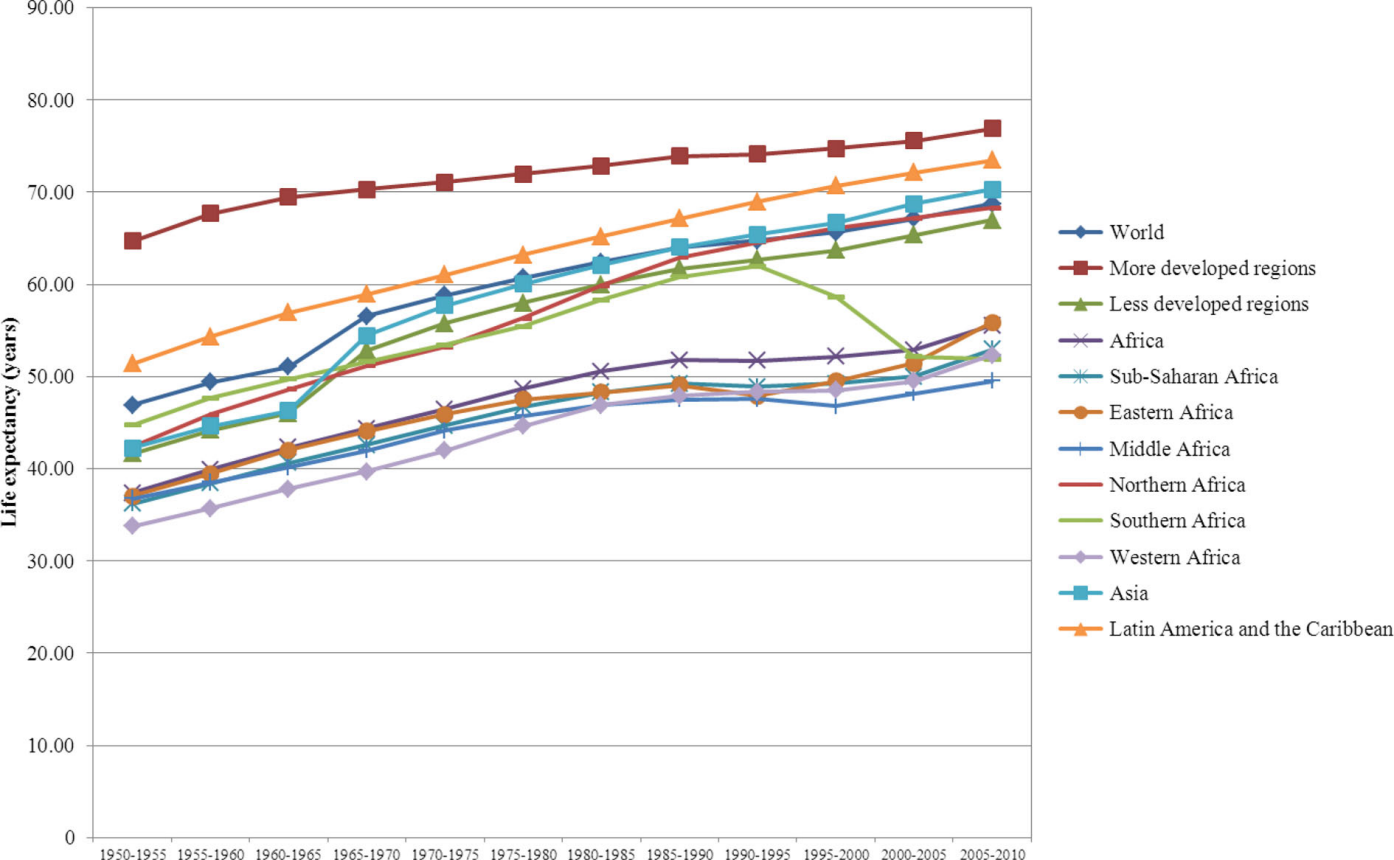
Life Expectancy at Birth for Africa and its regions, and other regions of the World, 1950–2010.

There are four emerging patterns, as illustrated in Fig. 6 for both sexes combined to avoid clutter (separate figures for males and females are available upon request). First, life expectancy at birth has been consistently lower in SSA than in Northern Africa for both males and females. Second, among both sexes Western Africa had the lowest life expectancy in Africa from 1950 to 1955 though the mid-1980s, but since then Middle Africa has had the lowest. Third, while Southern Africa was the region of the African continent with the highest life expectancy at birth from 1950 to 1955 through 1985–1990, life expectancy at birth in this region declined by 6 years between 1990–1995 and 2005–2010; losses were more drastic for females (13 years) than for males (8 years), which resulted in a narrowing of the life expectancy gap between females and males from 7 years in 1990–1995 to only 2 years in 2005–2010. Fourth, Eastern Africa experienced a 1-year loss in life expectancy at birth between 1985–1990 and 1990–1995, which can be ascribed to a dramatic drop in life expectancy in one country: The Rwanda genocide led to a decline in life expectancy in Rwanda from 45 years in 1985–1990 to a historically low life expectancy of 24 years. This demonstrates how wars and civil conflicts – a fact of life in African countries since the independence years in the 1960s – play a noticeable role in mortality patterns and life expectancy prospects in the continent and in SSA in particular.

Table 5 present the sex differentials in life expectancies at birth, at age 15 and at age 60 from 1950–1955 to 2005–2010, at four distinct periods of time, to capture possible period effects in these differentials.

**Table 5:** Sex differential (female–male) in life expectancy at birth, at age 15 and at age 60 by major areas, African regions and countries: 1950–2010

	F–M in 1950–1955			F–M in 1980–1985			F–M in the 1990–1995			F–M in 2005–2010		
	
Major areas, African regions and countries	e(0)	e(15)	e(60)	e(0)	e(15)	e(60)	e(0)	e(15)	e(60)	e(0)	e(15)	e(60)
World	2.00	3.07	2.02	4.53	4.73	3.05	4.63	4.85	3.15	4.48	4.58	2.98
More developed regions	5.10	4.53	2.50	7.52	7.23	4.21	7.78	7.57	4.31	7.04	6.93	3.95
Less developed regions	0.76	1.83	1.43	3.22	3.32	2.05	3.40	3.56	2.23	3.57	3.63	2.29
Sub-Saharan Africa	2.54	2.09	0.96	3.03	2.57	1.24	2.88	2.37	1.29	1.78	1.22	1.33
Africa	2.34	2.26	1.05	3.16	2.79	1.46	3.07	2.63	1.53	2.15	1.68	1.57
Eastern Africa	2.80	2.31	1.01	3.13	2.60	1.13	2.82	2.21	1.19	1.91	1.07	1.37
Burundi	3.13	2.50	1.05	3.53	2.76	1.18	3.02	2.31	1.13	2.97	2.02	1.21
Comoros	2.50	2.07	1.23	4.00	2.93	1.66	3.67	2.81	1.65	2.67	2.35	1.56
Djibouti	2.66	2.27	0.97	3.09	2.57	1.12	3.25	2.83	1.15	2.94	2.40	1.19
Eritrea	3.96	2.60	2.42	3.99	2.77	2.44	4.47	3.65	2.75	4.62	4.37	3.30
Ethiopia	2.59	2.18	0.91	2.90	2.41	1.04	2.64	2.09	1.10	2.24	1.36	1.21
Kenya	3.63	2.78	1.18	3.75	2.93	1.25	3.61	2.80	1.35	2.43	1.39	1.41
Madagascar	2.03	1.47	0.53	2.07	2.12	0.97	2.60	2.35	1.05	2.85	2.27	1.22
Malawi	0.92	1.22	0.52	2.00	2.10	0.97	2.18	2.01	1.20	−0.03	−0.53	1.77
Mauritius	2.47	3.16	2.92	7.30	7.12	4.49	7.42	7.14	4.15	6.86	6.71	3.90
Mayotte	5.68	6.21	4.40	8.09	7.80	4.22	7.92	7.70	4.20	7.40	7.28	4.14
Mozambique	2.40	2.05	0.85	3.10	2.51	1.07	2.99	2.48	1.07	2.25	1.09	1.51
Réunion	5.68	6.21	4.40	8.09	7.80	4.22	7.92	7.70	4.20	7.40	7.28	4.14
Rwanda	3.13	2.51	1.06	3.28	2.87	1.13	3.37	2.29	1.07	2.59	2.02	1.16
Seychelles	4.43	4.18	1.62	7.90	7.38	3.92	10.04	9.62	5.16	9.22	8.81	4.81
Somalia	2.96	2.37	0.98	3.09	2.51	1.07	3.04	2.54	1.09	3.15	2.63	1.16
South Sudan	2.73	2.21	0.90	2.82	2.34	0.99	2.76	2.30	1.04	1.99	1.46	1.07
Uganda	3.13	2.51	1.06	3.13	2.66	1.11	2.40	1.81	1.10	1.06	0.06	1.16
Tanzania	3.34	2.63	1.11	3.24	2.66	1.15	2.87	2.14	1.26	1.62	0.83	1.29
Zambia	3.00	2.45	1.05	3.39	2.89	1.22	1.20	0.39	1.14	1.26	0.59	1.14
Zimbabwe	3.10	2.56	1.12	3.71	2.97	1.26	3.06	2.51	1.63	−1.03	−1.99	1.72
Middle Africa	3.12	2.53	1.07	2.92	2.45	1.05	3.07	2.50	1.12	2.79	2.11	1.15
Angola	2.88	2.35	0.97	2.93	2.40	1.02	3.86	2.89	1.24	2.83	2.16	1.15
Cameroon	2.66	2.25	0.96	3.07	2.57	1.12	2.93	2.42	1.12	1.86	1.15	1.12
Central African Republic	2.88	2.33	0.97	4.94	3.63	1.38	4.49	3.31	1.58	3.32	1.67	1.52
Chad	5.50	3.74	1.52	2.23	2.10	0.91	2.33	2.02	1.02	1.48	1.24	0.98
Congo	1.79	1.83	0.81	2.98	2.59	1.10	3.03	2.29	1.26	2.51	1.73	1.19
DR Congo	2.82	2.34	0.99	2.83	2.38	1.03	2.85	2.41	1.05	3.31	2.65	1.16
Equatorial Guinea	2.98	2.39	0.99	3.34	2.70	1.12	3.19	2.55	1.13	2.66	2.05	1.16
Gabon	3.08	2.48	1.04	3.19	2.63	1.14	3.00	2.44	1.16	2.10	1.46	1.20
Sao Tome and Principe	2.89	2.41	1.04	3.68	2.91	1.26	3.64	2.89	1.26	3.80	2.98	1.31
Northern Africa	1.88	3.44	1.75	3.75	3.69	2.17	4.03	3.76	2.23	3.95	3.66	2.25
Algeria	1.25	1.59	0.77	2.93	2.70	1.61	3.20	2.82	1.78	3.08	2.73	1.85
Egypt	1.04	5.25	2.80	4.53	4.97	2.84	4.79	4.74	2.72	4.65	4.44	2.68
Libya	2.25	0.92	0.26	3.63	3.11	1.76	3.36	3.00	1.86	3.81	3.64	2.82
Morocco	3.43	2.75	1.20	3.05	2.73	1.58	3.40	2.92	1.79	3.40	2.91	1.89
Sudan	2.84	2.39	1.03	2.93	2.48	1.08	3.13	2.78	1.15	3.46	3.16	1.16
Tunisia	2.13	1.31	0.31	3.15	2.61	1.85	4.59	4.20	3.07	4.74	4.72	3.47
Western Sahara	3.09	2.33	1.24	3.26	2.55	1.55	3.30	2.66	1.64	3.83	3.03	1.85
Southern Africa	2.23	1.75	2.13	6.56	5.61	3.43	6.82	6.00	3.90	2.40	1.39	4.07
Botswana	3.90	2.90	1.58	4.15	3.18	1.79	5.19	4.51	2.52	−0.31	−1.06	3.40
Lesotho	2.63	2.16	1.27	2.60	2.26	1.49	2.91	2.52	1.65	0.49	−0.47	1.71
Namibia	4.60	3.20	1.59	4.56	3.32	1.82	4.83	3.49	1.99	5.65	4.59	2.39
South Africa	2.00	1.56	2.21	6.90	5.89	3.68	7.21	6.35	4.19	2.58	1.55	4.32
Swaziland	4.00	2.88	1.49	3.88	2.92	1.69	2.94	2.33	1.67	−0.51	−1.56	1.79
Western Africa	2.13	1.75	0.56	2.26	1.87	0.87	2.17	1.79	0.86	1.01	0.93	0.62
Benin	0.30	−1.29	−0.49	6.42	4.38	1.71	4.58	2.46	1.28	2.74	1.97	1.11
Burkina Faso	1.98	2.74	0.85	2.36	2.32	1.11	2.36	2.28	1.07	1.16	1.02	0.72
Cape Verde	2.21	2.08	1.33	2.54	2.30	1.57	6.80	4.67	2.40	7.89	6.50	4.30
Côte d'Ivoire	1.84	1.88	0.80	4.01	3.01	1.27	3.86	2.94	1.25	1.54	1.07	0.52
Gambia	2.35	1.89	0.53	2.56	2.06	0.95	2.57	2.11	1.04	2.55	2.18	1.14
Ghana	0.49	0.88	0.41	2.34	1.99	1.00	2.32	2.04	1.11	1.71	1.41	0.91
Guinea	2.26	1.76	0.54	2.40	2.01	0.89	1.47	1.12	0.80	1.37	1.51	0.87
Guinea-Bissau	1.93	1.62	0.63	3.84	2.84	1.20	4.40	3.23	1.42	2.88	1.66	0.93
Liberia	5.17	3.49	1.12	3.70	2.68	1.18	4.01	3.09	1.35	1.74	1.45	0.89
Mali	1.41	1.88	0.48	1.72	1.59	0.76	−0.29	−0.80	0.08	−0.43	−0.88	0.09
Mauritania	0.18	0.69	0.27	2.66	2.13	1.15	2.73	2.09	1.20	2.97	2.20	1.29
Niger	−0.12	0.47	0.12	0.21	0.46	0.40	0.52	0.51	0.55	0.17	0.22	0.49
Nigeria	2.65	1.97	0.63	2.09	1.75	0.81	2.07	1.74	0.81	0.58	0.57	0.41
Senegal	1.30	1.26	0.40	2.73	2.38	1.15	3.20	2.71	1.41	2.82	2.37	1.33
Sierra Leone	3.49	−0.24	−0.19	0.43	−1.15	−0.35	1.48	0.57	0.10	0.30	−0.17	0.01
Togo	1.63	1.43	0.46	3.67	2.88	1.36	1.73	0.95	0.73	1.46	0.97	0.65

Source: Author's calculations based on online datasets from United Nations, Department of Economic and Social Affairs, Population Division (2013). World Population Prospects: The 2012 Revision, DVD Edition. New York: United Nations.

Notes: e(0) = life expectancy at birth (in years); e(15) = life expectancy at age 15 (in years); e(60) = life expectancy at age 60 (in years).

Just as the reasons for health improvements represent an important area of investigation in health transition research, so do sex differentials in life expectancies. The epidemiological and health transitions suppose that because infants and children of both genders, adolescents and young adult women were at greatest risk of premature death from infectious diseases during the pre-transitional period, reduction in the incidence of these conditions will result in an improvement in life expectancy, initially due to early reduction of infant and maternal mortality. A number of findings emerge from Table 5. There is female advantage in life expectancy at birth and at age 15 in all major regions considered and in all African countries with the exception of Malawi, Zimbabwe, Botswana, Swaziland and Mali in 2005–2010. One plausible explanation is the excess female AIDS-related mortality or harmful reproductive practices for female children and adolescent girls in these countries (66, 138). Deleterious reproductive health practices or differential treatment may also account for the female disadvantage in life expectancy at ages 15 and 60 for Benin, Mali and Sierra Leone in earlier periods than the 1990s and beyond when the possibility of AIDS-reducing life expectancy cannot be ruled out in some African countries with high HIV infection rates. Finally, there is robust female advantage in life expectancy at age 60 for all regions and countries over time, but Sierra Leone up to the 1980–1985 period. Overall, there are some inconsistent patterns for a handful of countries, but a general female advantage in life expectancy at birth, at age 15 and age 60 for the vast majority of countries.

In Table 6, the patterns of trends in total fertility and life expectancies between 1950–1955 and 2005–2010 for African regions (and SSA notably) are compared with the other world regions.

**Table 6:** Global context of patterns of trends in total fertility (TF) and life expectancy at birth (e(0)), at age 15 (e(15)) and age 60 (e(60)) in Africa

	TF: 1950–55		TF: 2005–10		e(0): 1950–55		e(0): 2005–10		e(15): 1950–55		e(15): 2005–10		e(60) 1950–55		e(60): 2005–10	
	
Major regions	(a)	(b)	(a)	(b)	(c)	(d)	(c)	(d)	(c)	(d)	(c)	(d)	(c)	(d)	(c)	(d)
World	4.97	1.57	2.53	2.86	46.91	−10.69	68.72	−15.78	46.94	−6.08	58.80	−11.31	14.16	−2.14	19.73	−4.27
More developed regions	2.83	3.70	1.66	3.73	64.67	−28.44	76.90	−23.96	55.65	−14.79	62.60	−15.11	16.84	−4.83	22.07	−6.61
Less developed regions	6.08	0.45	2.69	2.70	41.62	−5.40	66.96	−14.02	42.97	−2.11	57.43	−9.94	12.35	−0.34	18.54	−3.08
Sub − Saharan Africa	6.53	–	5.39	–	36.23	–	52.94	–	40.86	–	47.49	–	12.01	–	15.46	–
Africa	6.60	−0.06	4.88	0.51	37.38	−1.15	55.55	−2.61	42.43	−1.57	49.56	−2.08	12.52	−0.51	15.98	−0.52
Eastern Africa	7.01	−0.48	5.38	0.01	37.03	−0.80	55.85	−2.91	41.98	−1.12	48.80	−1.31	12.75	−0.74	17.02	−1.56
Middle Africa	5.99	0.54	6.17	−0.78	36.77	−0.54	49.53	3.42	41.96	−1.09	47.54	−0.05	12.79	−0.78	15.42	0.04
Northern Africa	6.81	−0.27	3.07	2.32	42.36	−6.13	68.32	−15.37	49.00	−8.14	57.32	−9.83	14.66	−2.65	17.56	−2.10
Southern Africa	6.28	0.25	2.64	2.75	44.74	−8.52	51.89	1.05	40.91	−0.05	42.06	5.43	11.75	0.26	15.26	0.20
Western Africa	6.35	0.18	5.73	−0.34	33.75	2.48	52.32	0.62	39.56	1.31	47.77	−0.28	11.19	0.82	14.06	1.40
Asia	5.83	0.71	2.25	3.14	42.24	−6.02	70.28	−17.34	42.96	−2.10	59.16	−11.67	12.27	−0.26	19.12	−3.66
Eastern Asia	5.60	0.93	1.61	3.78	46.21	−9.99	75.52	−22.58	44.19	−3.33	62.24	−14.75	11.62	0.39	20.65	−5.19
South-Central Asia	6.02	0.51	2.72	2.67	37.43	−1.21	65.53	−12.59	40.62	0.24	55.70	−8.21	12.68	−0.66	17.04	−1.58
Central Asia	5.23	1.30	2.67	2.72	54.51	−18.29	66.28	−13.34	51.45	−10.59	55.48	−7.99	15.28	−3.27	17.17	−1.71
Southern Asia	6.05	0.48	2.72	2.67	36.98	−0.75	65.51	−12.56	40.27	0.59	55.70	−8.21	12.55	−0.53	17.04	−1.58
South-Eastern Asia	5.92	0.62	2.35	3.04	43.98	−7.76	70.34	−17.39	45.01	−4.15	58.23	−10.74	13.95	−1.93	18.68	−3.22
Western Asia	6.32	0.22	2.92	2.47	42.61	−6.38	72.24	−19.30	47.20	−6.33	60.13	−12.65	14.10	−2.08	19.33	−3.87
Europe	2.67	3.87	1.54	3.85	63.59	−27.36	75.28	−22.34	55.71	−14.85	61.01	−13.52	16.79	−4.77	21.08	−5.62
Eastern Europe	2.91	3.63	1.41	3.98	60.33	−24.11	69.52	−16.58	54.46	−13.60	55.55	−8.06	16.54	−4.53	17.99	−2.53
Northern Europe	2.32	4.21	1.86	3.53	68.76	−32.54	79.11	−26.17	57.07	−16.21	64.62	−17.13	16.99	− 4.97	22.65	−7.19
Southern Europe	2.68	3.86	1.43	3.96	63.45	−27.22	79.92	−26.98	55.92	−15.06	65.48	−18.00	16.73	−4.72	23.15	−7.69
Western Europe	2.39	4.15	1.64	3.75	67.72	−31.50	80.21	−27.27	56.76	−15.90	65.66	−18.17	16.95	−4.94	23.46	−8.00
Latin America & Caribbean	5.86	0.67	2.30	3.09	51.37	−15.14	73.45	−20.51	49.62	−8.76	60.86	−13.37	15.36	−3.35	21.27	−5.81
Caribbean	5.27	1.27	2.37	3.02	52.05	−15.83	71.22	−18.28	50.21	−9.35	60.06	−12.57	15.57	−3.55	21.05	−5.59
Central America	6.73	−0.20	2.56	2.83	49.13	−12.91	75.26	−22.32	48.49	−7.63	62.42	−14.93	15.27	−3.26	22.00	−6.54
South America	5.66	0.87	2.19	3.20	52.10	−15.87	73.03	−20.09	49.92	−9.06	60.42	−12.93	15.37	−3.36	21.06	−5.60
Northern America	3.35	3.18	2.02	3.37	68.59	−32.36	78.36	−25.42	56.54	−15.68	64.08	−16.59	17.44	−5.42	22.88	−7.42
Oceania	3.84	2.70	2.47	2.92	60.44	−24.22	76.84	−23.90	52.49	−11.63	64.36	−16.87	16.35	−4.33	23.33	−7.87

Source: Author's calculations using online databases from United Nations, Department of Economic and Social Affairs, Population Division (2013). World Population Prospects: The 2012 Revision, DVD Edition. New York: United Nations.

Notes: SSA = sub-Saharan Africa; (a) = total fertility (TF); (b) = TF(SSA) – TF(Region); (c) = life expectancy (e); (d) = e(SSA) – e(Region).

With respect to fertility trends, it is obvious that ‘African exceptionalism’ is undeniable in the sense that fertility decline has been slowest in SSA and its regions, compared to the rest of regions.

People around the world, including all its major regions, continents, and countries, have enjoyed significant increases in life expectancies over the last 60 years. This has largely been the result of medical advances directed against infectious and parasitic diseases in developing countries along with the cardiovascular revolution in the developed world, which together have been pushing forward the threshold conjectured by Omran's epidemiological transition. Table 6 demonstrates that the exception is Africa (and SSA in particular), which had a life expectancy at birth of less than 40 years in 1950–1955 and has ever since had the world's lowest life expectancy. In fact, the health disparities in terms of life expectancy have remained or have widened consistently over the last 60 years between sub-Saharan African regions and all the other regions of the world, with respect to gap in life expectancy at birth, at age 15 and at age 60. Moreover, whereas all other of the world's regions have enjoyed uninterrupted increases in life expectancy, SSA and its regions and countries had a peak in life expectancy in the early 1990s and by 2010 some sub-Saharan African countries, such as Zimbabwe, were experiencing pre-1950 life expectancy levels. The stagnation in gains and reversals in trends in life expectancy for sub-Saharan African countries in the 1990s mainly reflect increasing mortality from HIV/AIDS. The consistent evidence from Table 6 points towards movements away from predicted trajectories of life expectancies and fertility rates inferred by the demographic, epidemiological or health transitions as predictive models for describing the historical experiences of population health change in African regions and countries over the past 60 years.

## Discussion

The demographic transition and epidemiological transition are compelling frameworks which have provided an accurate description of historical experiences regarding the secular declines in mortality and fertility and the associated changes in patterns of disease and causes of death through the 1950s in today's developed countries. New forms of demographic change (88, 89) and epidemiological landscape (84, 85) have emerged in the developed world around the 1960s and 1970s, and new population and health developments have been occurring in less developed countries too (12–16, 29, 45, 58, 66–70, 74, 80, 140). The extent to which these historical experiences from developed countries and predictions derived from them could inform the demographic and epidemiological changes in developing countries have been called into question from the beginning, and remains an empirical question to which this study has provided a systematic answer.

This study has shown that the demographic, epidemiological, and health landscapes of the African continent are significantly different from those experienced by the now-developed countries of Europe and North America during their demographic and epidemiological transitions. The evidence clearly shows that, with the notable exception of island African countries such as Mauritius and probably some northern African nations, over the past 60 years African countries have not embarked on any evidence-based shift from one epidemiological regime to another and have not seen demographic changes and health improvements, as would be predicted from the demographic, epidemiological, and health transition frameworks or from the global trends when they are compared to other developing countries outside of Africa. Instead, the expected steady improvement in health and disease prevention has been short-circuited by PI, a lack of social consciousness, and health-care systems that are too ill-prepared and underfunded to deal effectively with the challenges of increasingly co-occurring communicable and NCDs in national populations. It seems certain that the epidemiological and demographic changes in Africa over the past six decades would have been completely different – and with a better outlook – if there had been 60 years of continuous social and political stability.

Africa is a continent of uncertainties, emergencies, and humanitarian crises against a backdrop of cultural, economic, political and geographic diversities, a situation that has created inertia in population and health development. As a consequence, the health and disease patterns in much of the continent remain characterized by the predominance of communicable diseases, with 65% of all deaths caused by diseases amenable to interventions. The 1990s saw losses in life expectancy for many national populations in Southern and Eastern Africa, and there is mounting evidence indicating that Middle Africa may undergo a similar course, triggered by the spread of the HIV/AIDS epidemic (141–145). The resulting interruptions in life expectancy improvements in many African countries are unprecedented in human history.

Before the 1970s, there were almost no examples of long-term reversals in mortality, with the obvious exceptions of those caused by war and famine. Reflecting this, many of the classic analyses of the 1970s that examined long-term demographic and epidemiological trends concluded that while further significant gains in longevity in countries with low mortality were unlikely, death rates in countries with the high mortality typical of African countries would fall, resulting in a worldwide convergence in mortality (2, 146). However, since the 1990s all five countries of Southern Africa, three countries in Middle Africa (Cameroon, Central African Republic and Chad), Burundi and Kenya in Eastern Africa, Côte d'Ivoire in West Africa, and several countries of Eastern Europe have experienced reversals in mortality trends and losses in life expectancy at birth. Most recently, from 1995 onwards, the mortality patterns has become much more diffuse, with several countries in Southern Africa, Eastern Africa, and Middle Africa experiencing stagnation or deceleration in improvements in life expectancy and declines in life expectancy at birth. These reversals have occurred in a context of pervasive civil wars and social unrest, PI, substandard public expenditures on health, precarious socioeconomic conditions for large percentages of the population who are mainly employed in the informal sector with no social security, the HIV/AIDS epidemic and its co-morbidities, an increasing prevalence of infectious diseases, and raging food insecurity in this historically agricultural continent.

By and large, the conjectured linkages between mortality, fertility, and population growth find little empirical support in much of Africa, calling into question the basic premise of the transition approach embodied in the demographic, epidemiological, and health transition models. Despite signs of an onset of fertility decline in a handful of African countries (39, 68, 147–149), the widening gap between fertility and mortality patterns within and across countries combined with the enduring prevalence of infectious diseases in the continent suggests that a new and different perspective is needed for understanding health and disease trends in Africa.

The lack of available and adequate historical epidemiological data on disease, morbidity and mortality by cause of death and the influences on them remain a major obstacle for analyzing health and disease patterns in Africa. The cause-of-death data in Africa remain very scarce, and there is an urgent need for collecting and analyzing such data. The relative weightings of the various sources of morbidity and mortality and the ways in which these change over time have great significance for the planning and provision of health and other human services. The lack of suitable longitudinal data on health and disease patterns is probably the major limiting factor for local and national studies for many African countries.

Our study indicates that, given that most populations in Africa live under continuous co-occurrence of infectious and non-infectious diseases origin with various degrees of chronicity, any useful study of the health and disease patterns in Africa will be theoretically, programmatically, and empirically limited unless morbidity and mortality are linked within a health and disease continuum framework. This is an area that deserves future research. In those African countries for which studies have been carried out, the disease and mortality patterns indicate that both morbidity and mortality have been rising over time for major diseases often occurring concurrently (44, 50, 51, 140). With an increasing dual burden of disease in SSA, our understanding of the associations between diseases will become of increased public health importance. Research is also needed to develop effective approaches to reducing the frequency and health impact of the co-morbidities.

## Conclusion

Empirical evidence from 57 African countries substantiates that existing transition frameworks are not adequate for describing the patterns of demographic, epidemiological and health changes which have taken place in Africa over the last 60 years and beyond, nor are they predictive models of concurrent trajectories of population health resulting from historical and contemporary forces of change in the continent.

Prevailing frameworks of demographic, epidemiological, and health transitions as descriptive and explanatory tools are beyond reasonable doubt incomplete or irrelevant in the African context on conceptual, methodological, empirical, and policy grounds. Charting the course of health and disease patterns in African countries and regions through functional systems of continuous and sustainable national and local data collection enterprises will significantly reduce the burden of disease in these countries and will undoubtedly narrow the health and economic gap between the developed and developing worlds as well as among African regions and countries.

Omran (10, 11) acknowledged that the dynamics of the Western transition were closely related to the unique historical and circumstantial experience of the industrial and social revolution in the West during the last three centuries, and may not automatically be transferable to less developed countries. Despite substantial uncertainty about the extent of changes in levels and differentials in mortality statistics and patterns of causes of death prevailing in various groups of regional or national populations studied, it is obvious that population and health changes in Africa have so far deviated from the demographic, epidemiological and health transitions.

**Main findings**

- For research, planning and policy purposes, empirical evidence spanning at least the past 60 years substantiates that the demographic, epidemiological and health transition frameworks are inadequate for establishing a comprehensive representation of past and current situations at the regional, national and sub-national levels in the African context.
- Regional and national patterns of changes in mortality, fertility, population growth, and cause of death structure in Africa and other regions of the world between 1950 and 2010, highlight that many African countries distinctively undergo uncharted paths compared to historical experiences of Europe and North America.
- The demographic transition, epidemiological transition and health transition are irrelevant for predicting how demographic and epidemiological changes will play out across and within African countries and regions where changing contexts of health, disease and mortality patterns are embedded in significant uncertainties.

**Key messages for action**

- Upon scrutiny of the concepts of demographic transition, epidemiological transition and health transition, these concepts may not be used synonymously. There are significant differences between them regarding their level of specificity and generalizability, their descriptive and explanatory dimensions, their prognostic implications and their empirical soundness in low- and middle-income countries.
- Model-based estimates of health, disease and mortality statistics for African countries are at best conjectural in the vast majority of African societies where vital events, health conditions, disabilities, accidents and their risk factors are undetected. Limited data available to health planners to better pinpoint and address these problems often emanate from small-scale rural sites or are poorly recorded or preserved. National governments, the World Health Organization and pertinent United Nations agencies, the World Bank, international foundations, funding agencies along with researchers, should move from ceaseless pious vows to concerted actions aligned to resources in order to collect nationally representative data on measures of health and disease burden as well as their risk factors in Africa for research, planning and policy.
- A new perspective embodying broadly suitable theoretical guidance is needed. Such perspective will be used to organize data collection, to develop models of demographic and epidemiological changes that can serve as a basis of hypotheses to be tested, and to inform a well-grounded understanding of how demographic and epidemiological profiles have changed or can be expected to change in the future. Such understanding will help the institutional capacity development as well as the planning and development of health systems and health policies in typically heterogeneous African societies.
